# Establishing *Physalis* as a *Solanaceae* model system enables genetic reevaluation of the inflated calyx syndrome

**DOI:** 10.1093/plcell/koac305

**Published:** 2022-10-21

**Authors:** Jia He, Michael Alonge, Srividya Ramakrishnan, Matthias Benoit, Sebastian Soyk, Nathan T Reem, Anat Hendelman, Joyce Van Eck, Michael C Schatz, Zachary B Lippman

**Affiliations:** Cold Spring Harbor Laboratory, Cold Spring Harbor, New York 11724, USA; Howard Hughes Medical Institute, Cold Spring Harbor Laboratory, Cold Spring Harbor, New York 11724, USA; Department of Computer Science, Johns Hopkins University, Baltimore, Maryland 21218, USA; Department of Computer Science, Johns Hopkins University, Baltimore, Maryland 21218, USA; Cold Spring Harbor Laboratory, Cold Spring Harbor, New York 11724, USA; Howard Hughes Medical Institute, Cold Spring Harbor Laboratory, Cold Spring Harbor, New York 11724, USA; Cold Spring Harbor Laboratory, Cold Spring Harbor, New York 11724, USA; Boyce Thompson Institute, Ithaca, New York 14853, USA; Cold Spring Harbor Laboratory, Cold Spring Harbor, New York 11724, USA; Boyce Thompson Institute, Ithaca, New York 14853, USA; Plant Breeding and Genetics Section, School of Integrative Plant Science, Cornell University, Ithaca, New York 14853, USA; Cold Spring Harbor Laboratory, Cold Spring Harbor, New York 11724, USA; Department of Computer Science, Johns Hopkins University, Baltimore, Maryland 21218, USA; Department of Biology, Johns Hopkins University, Baltimore, Maryland 21218, USA; Cold Spring Harbor Laboratory, Cold Spring Harbor, New York 11724, USA; Howard Hughes Medical Institute, Cold Spring Harbor Laboratory, Cold Spring Harbor, New York 11724, USA

## Abstract

The highly diverse *Solanaceae* family contains several widely studied models and crop species. Fully exploring, appreciating, and exploiting this diversity requires additional model systems. Particularly promising are orphan fruit crops in the genus *Physalis*, which occupy a key evolutionary position in the *Solanaceae* and capture understudied variation in traits such as inflorescence complexity, fruit ripening and metabolites, disease and insect resistance, self-compatibility, and most notable, the striking inflated calyx syndrome (ICS), an evolutionary novelty found across angiosperms where sepals grow exceptionally large to encapsulate fruits in a protective husk. We recently developed transformation and genome editing in *Physalis grisea* (groundcherry). However, to systematically explore and unlock the potential of this and related *Physalis* as genetic systems, high-quality genome assemblies are needed. Here, we present chromosome-scale references for *P. grisea* and its close relative *Physalis pruinosa* and use these resources to study natural and engineered variations in floral traits. We first rapidly identified a natural structural variant in a *bHLH* gene that causes petal color variation. Further, and against expectations, we found that CRISPR–Cas9-targeted mutagenesis of 11 MADS-box genes, including purported essential regulators of ICS, had no effect on inflation. In a forward genetics screen, we identified *huskless*, which lacks ICS due to mutation of an *AP2-like* gene that causes sepals and petals to merge into a single whorl of mixed identity. These resources and findings elevate *Physalis* to a new *Solanaceae* model system and establish a paradigm in the search for factors driving ICS.

## Teaser

Genomics and genetics resources establish *Physalis* as a new *Solanaceae* reference system and enable reevaluation of the inflated calyx syndrome.

## Introduction

The *Solanaceae* family is one of the most important plant families in fundamental and applied research not only due to its remarkable morphological and ecological diversity but also due to its far-reaching economic value from its many members used as food crops, ornamentals, and sources of pharmaceuticals (Gebhardt, 2016; Shenstone et al., 2020; Añibarro-Ortega et al., 2022). The most studied *Solanaceae* include major food crops such as eggplant (*Solanum melongena*), pepper (*Capsicum annuum*), potato (*Solanum tuberosum*), and tomato (*Solanum lycopersicum*), in addition to the model species petunia (*Petunia hybrida*) and *Nicotiana benthamiana*. However, various species-specific limitations of the other taxa have made tomato a preferred model for many studies, as it has a full suite of genetic and genomic resources that enable maximal biological discovery and translation to agriculture.

Developing new *Solanaceae* model systems that equal the utility of tomato is essential to study incompletely explored diversity, including traits of economic importance. The most challenging is identifying potential systems with noteworthy comparative and species-specific variation that, critically, can be dissected by efficient forward and reverse genetics that is enabled by tractable genomics, genome editing, and cultivation. We previously identified species in the genus *Physalis* as promising in all these aspects (Lemmon et al., 2018). This genus includes orphan crops such as tomatillo (*Physalis philadelphica* and *Physalis ixocarpa*), goldenberry (*Physalis peruviana*), and groundcherry (*Physalis grisea* and *Physalis pruinosa*), and many other species that yield edible fruits or are grown as ornamentals.

*Physalis* occupies a key phylogenetic position that complements other *Solanaceae* models. It is a representative genus of Physaleae, an under-studied Solanaceae tribe that has the most genera in the family (Zamora-Tavares et al., 2016; Deanna et al., 2019; Pretz and Deanna, 2020), and diverged from established *Solanum* model systems about 19 million years ago (Ma; Särkinen et al., 2013). In addition, recently discovered Physaloid fruiting fossils dated to about 52 Ma pushed back the evolutionary timing of *Solanaceae* divergence from other taxa considerably (Wilf et al., 2017; Deanna et al., 2020). Thus, *Physalis* has great potential to analyze diversification over long evolutionary distances in comparative studies within the *Solanaceae*. Moreover, *Physalis* species show substantial variation in developmental and molecular traits, including inflorescence complexity, secondary metabolism, and disease resistance (Baumann and Meier, 1993; Whitson, 2012; Park et al., 2014; Zhang and Tong, 2016; Huang et al., 2020), providing additional avenues for discovery. However, the most conspicuous and impressive feature of *Physalis*, also found in other angiosperms, is the inflated calyx syndrome (ICS), a remarkable evolutionary novelty where sepals grow excessively large after fertilization to form balloon-like husks that encapsulate fruits (He et al., 2004; Wilf et al., 2017).

Dissecting the evolutionary and mechanistic origins of morphological novelties is a fundamental goal in biology (Muller and Wagner, 1991; Shubin et al., 2009), and it is not surprising that botanists and evolutionary biologists have long been fascinated by ICS (He et al., 2004; Waterfall and Umaldy, 1958; Wilf et al., 2017). Though *Physalis* has historically lacked molecular and functional genetics tools, studies on ICS over the last few decades have suggested a central role for two MADS-box genes, including an ortholog of one gene in potato, *StMADS16* (an ortholog of *Arabidopsis thaliana AGAMOUS-LIKE 24*), which causes leaf-like sepals when overexpressed in other *Solanaceae* (He et al., 2004). Prompted by this observation, supportive molecular and functional genetic data generated within *Physalis* suggested that heterotopic expression of the *StMADS16* ortholog *MPF2* was key to the evolution of ICS. Later studies suggested this essential role emerged from modified cis*-*regulatory control of *MPF2* by the *euAP1-like* gene *MPF3* (He and Saedler, 2005; Zhao et al., 2013).

A recent genome of *Physalis floridana* and additional functional work suggested that loss of another MADS-box gene, *MBP21/JOINTLESS-2* (*J2*), a member of the *SEPALLATA4* (*SEP4*) clade, was also critical, and seemingly reinforced an additional conclusion that fertilization is an integral physiological driver of ICS (Lu et al., 2021). The proposed role of fertility and previous findings that flower-specific *MPF2* expression is ancestral to ICS suggested this trait may have been lost during evolution (He and Saedler, 2007; Hu and Saedler, 2007). However, a recent deeply sampled taxonomic study showed that, although being invariantly present in a large monophyletic clade such as *Physalis* subgenus *Rydbergi*, ICS was gained multiple times throughout the tribe of Physalideae in a stepwise and directional manner, from noninflation to enlarged sepals appressed to the fruit (accrescent–appressed), and finally to an inflated calyx (Deanna et al., 2019). These findings, along with independent emergence of ICS in other angiosperms (Deanna et al., 2019), may indicate that there is a deeper genetic and molecular complexity behind ICS, determined by factors besides *MPF2* and other proposed *MADS-box* genes (Hu and Saedler, 2007; Deanna et al., 2019).

Outstanding questions regarding ICS and our broad interest in *Solanaceae* biology and agriculture led us several years ago to begin establishing *Physalis* as a new model system. We developed efficient *Agrobacterium*-mediated transformation and CRISPR–Cas9 genome editing in the diploid groundcherry species *P. grisea*, and demonstrated the utility of these tools by mutating orthologs of tomato domestication genes in groundcherry to improve productivity traits (Lemmon et al., 2018; Swartwood and van Eck, 2019). More recently, *P. grisea* was critical in revealing pleiotropic functions of an ancient homeobox gene, and in dissecting the evolution of redundancy between duplicated signaling peptide genes controlling stem cell proliferation in the *Solanaceae* (Hendelman et al., 2021; Kwon et al., 2022). However, high-quality reference genomes of *P. grisea* and other species have been lacking, and are needed to promote the full potential and deployment of this system as has been achieved in tomato. Here, we report high-quality chromosome-scale genomes for *P. grisea* and its close relative *P. pruinosa.* We demonstrate the power of these resources in enabling forward and reverse genetics by revealing multiple genotype-to-phenotype relationships in floral development, including ICS. Our work establishes *Physalis* as a new *Solanaceae* reference system that can advance comprehensive studies of long-standing and emerging biological questions within and beyond the genus.

## Results

### Chromosome-scale reference genomes of *P. grisea* and *P. pruinosa*

**Figure 1:**
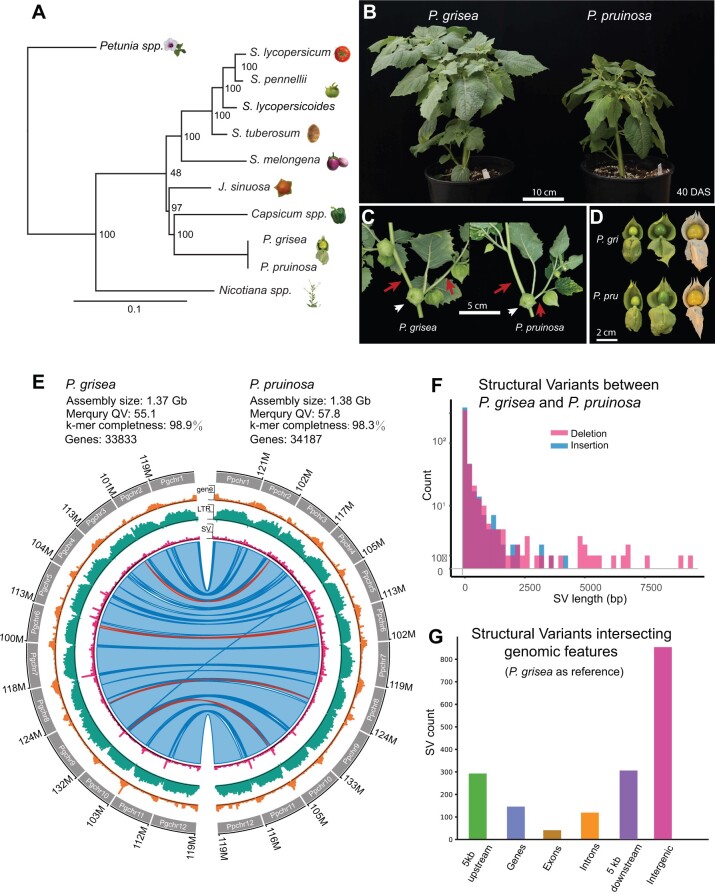
Reference-quality genome assemblies of *P. grisea* and *P. pruinosa.* A, Phylogeny of selected *Solanaceae* species based on the 20 most conserved protein sequences (see “Materials and methods”). B, Whole plant images of *P. grisea* and *P. pruinosa* 40 days after sowing in greenhouse conditions. Bar = 10 cm. C, Sympodial shoot architectures of *P. grisea* and *P. pruinosa*. Quantification of internode lengths is in Supplemental Data Set 1. Bar = 5 cm. D, Images of *P. grisea* and *P. pruinosa* calyces and fruits at different stages of development. Husks were manually opened to show fruits. Bar = 2 cm. E, Circos plots comparing *P. grisea* and *P. pruinosa* genomes. Circos quantitative tracks are summed in 100-kbp windows and show the number of genes (lower tick = 0, middle tick = 25, higher tick = 49), LTR retrotransposons (lower tick = 0, middle tick = 102, higher tick = 204) and SVs (lower tick = 0, middle tick = 4, higher tick = 9). The inner ribbon track shows whole genome alignments, with blue indicating forward-strand alignments and red indicating reverse-strand alignments (inversions). Darker colors indicate alignment boundaries. F, Distribution of deletion and insertion SVs between 30 bp and 10 kbp from *P. pruinosa* compared to *P. grisea*, summed in 200-bp windows. G, Counts of SVs intersecting genomic features, comparing *P. pruinosa* to *P. grisea*.

Among *Solanaceae* genera*, Physalis* is more closely related to *Capsicum* (pepper) than *Solanum* (eggplant, potato, and tomato) (Figure 1A). Chinese lantern (*Alkekengi officinarum*, closely related to *Physalis*), tomatillo (*P. philadelphica* and *P. ixocarpa*), and many other *Physalis* orphan crops are self-incompatible, large plants with tetraploid genomes, making them challenging to develop into model systems. In contrast, the groundcherry species *P. grisea*, *P. pruinosa*, and close relatives have reasonable genome sizes (estimated ∼1–2 Gb), are diploid, self- and cross-compatible, have rapid generation times (first mature fruit 66–70 days after sowing), and are easy to grow and manage in both greenhouses and fields. The taxonomy and naming of *Physalis* species have a convoluted past that was recently clarified (Pretz and Deanna, 2020). *Physalis pruinosa* was initially designated to describe *Physalis* in the northeastern USA, showing erect or prostrate growth with large, thick, and coarsely sinuate–dentate leaves (Rydberg, 1896). A revision of *Physalis* in the last century proposed *P. pubescens* var. *grisea* to differentiate species included in *P. pruinosa* (Waterfall and Umaldy, 1958). Additional species were then identified (Waterfall, 1967), and *P. pubescens* var. *grisea* was ultimately recognized as a separate species, *P. grisea* (Martínez, 1993; Pretz and Deanna, 2020).

As *P. grisea* and *P. pruinosa* are closely related, they share similar vegetative and reproductive shoot and organ morphologies, including inflated calyxes encapsulating fruits of similar size, shape, and color (Figure 1, B–D). Their primary shoots terminate in a single flower inflorescence after five to six leaves, and new shoots emerge according to the sympodial growth habit that is characteristic of all *Solanaceae* (Lemmon et al., 2018). In *Physalis*, sympodial units comprise one leaf, one flower, and two axillary (sympodial) shoots (Figure 1C). A conspicuous feature distinguishing *P. pruinosa* from *P. grisea* is the absence of purple pigmentation on stems and petal nectar guides. *Physalis pruinosa* also has narrower leaves and a smaller stature due to shorter internodes (Figure 1, B and D; Supplemental Data Set 1).

Based on the features described, *P. grisea* and *P. pruinosa* are excellent candidates occupying a key phylogenetic position among *Solanaceae* model systems. We integrated PacBio high fidelity (HiFi) and Oxford Nanopore Technology (ONT) long-read sequencing to establish highly accurate and complete chromosome-scale genome assemblies for both species, with assembly sizes of 1.37 Gb for *P. grisea* and 1.38 Gb for *P. pruinosa* (Figure 1E). The *P. grisea* and *P. pruinosa* assemblies are the first *Physalis* genus reference-quality assemblies, demonstrating substantially improved contiguity, accuracy, and completeness compared to a recent *P. floridana* genome (Lu et al., 2021) (Supplemental Table S1). Specifically, the *P. floridana* genome has an error rate (errors/bp) of 3.83 × 10^−4^ and a contig N50 of 4.6 Mbp, whereas our assemblies produced substantially lower error rates of 3.09 × 10^−6^ (*P. grisea*) and 1.66 × 10^−6^ (*P. pruinosa*) and much higher contig N50s of 31.6 and 82.2 Mbp, respectively, with gapless assemblies of chromosomes 5 and 7 for *P. pruinosa*.

Based on RNA-sequencing data from vegetative and reproductive tissues ((Lemmon et al., 2018), and see “Materials and methods”), we annotated 33,833 and 34,187 genes in the *P. grisea* and *P. pruinosa* assemblies, respectively (Supplemental Table S2), with most genes concentrated at the ends of the 12 chromosomes, as was observed in other *Solanaceae* genomes (Xu et al., 2011; Sato et al., 2012; Kim et al., 2014; Wei et al., 2020) (Figure 1E, see “Materials and methods”). Both genomes are highly repetitive, with 79% of the sequence representing transposable elements, especially long terminal repeat (LTR) retrotransposons (Figure 1E). Comparing the two genomes, we observed nearly complete macrosynteny across all 12 chromosomes, consistent with the close relationship of these species, but also detected a few small-scale inversions and translocations (Figure 1E). Calling single-nucleotide polymorphisms (SNPs) using *P. pruinosa* Illumina short-read sequences against the *P. grisea* reference revealed 60,087 homozygous SNPs, with predicted high-impact changes (SNPeff, Cingolani et al., 2012) on 43 gene transcripts (Supplemental Tables S3 and S4). Despite the broad similarity of these genomes, we identified over 900 structural variants (SVs) between 30 bp and 10 kb in length, many of which intersect coding and putative cis-regulatory sequences (Figure 1, F and G; Supplemental Table S5; Supplemental Data Set S2). Some of these variants could explain phenotypic differences between *P. grisea* and *P. pruinosa*.

### An SV in the bHLH transcription factor gene *AN1* controls nectar guide color variation

**Figure 2:**
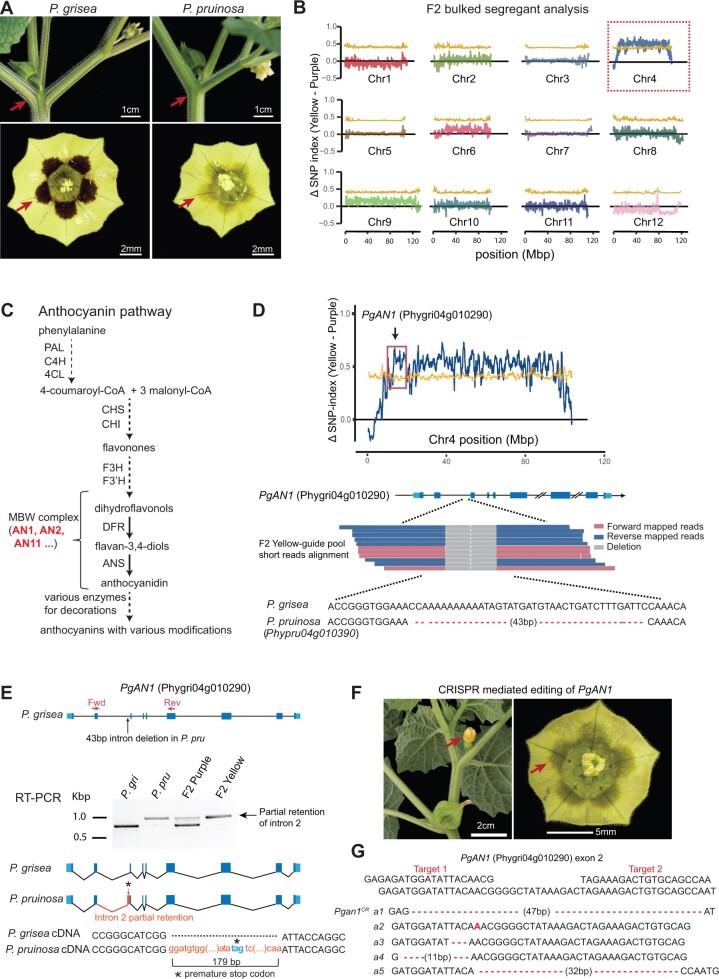
Loss of purple pigmentation in *P. pruinosa* is due to an intronic SV in the bHLH transcription factor gene *ANTHOCYANIN1*. A, Images showing the difference in pigmentation between *P. grisea* and *P. pruinosa*. Arrows point to purple (*P. grisea*) compared to yellow (*P. pruinosa*) pigmentation on stems and flowers. Top bars = 1 cm; bottom bars = 2 mm. B, Mapping by sequencing showing the ΔSNP-index across all twelve chromosomes using *P. grisea* as the reference, with SNP ratios between yellow-guide and the purple-guide pools from an interspecific F2 population. Yellow line: 95% confidence interval cut-offs of ΔSNP-index. C, Simplified pathway of anthocyanin biosynthesis based on data from petunia. Major transcriptional and enzymatic regulators are shown as abbreviations. PAL, Phenylalanine Ammonialyase; C4H, Cinnamate 4-Hydroxylase; 4CL, 4-Coumaroyl-CoA ligase; CHS, Chalcone Synthase; CHI, Chalcone Isomerase; F3H, Flavanone 3-hydroxylase; F3'H, Flavonoid 3'-hydroxylase; AN1, ANTHOCYANIN 1; AN2, ANTHOCYANIN 2; AN11, ANTHOCYANIN 11; DFR, Dihydroflavonol Reductase; ANS, Anthocyanin Synthase. Dashed lines indicate multiple steps condensed. Bold red font indicates components of the MYB-bHLH-WD40 complex that transcriptionally activates late biosynthetic genes. D, Top: The ΔSNP-index plot for chromosome 4. The black arrow points to the genomic location of the *AN1* candidate gene. Bottom: a composite of Illumina mapped-reads from *P. pruinosa* at the second intron of *AN1* showing a 43-bp deletion in all *PpAN1* (*Phypru04g010390*) sequences. In all gene models (including later figures), deep blue boxes, black lines, and light blue boxes represent exonic, intronic, and untranslated regions, respectively. E, Molecular consequences of the 43-bp intronic deletion in *PpAN1* revealed by RT-PCR and sequencing. Arrows indicate the forward and reverse RT-PCR primers. Longer amplicons and thus *AN1* transcripts from both the yellow-guide F2 bulk pool and *P. pruinosa* were identified by agarose gel electrophoresis. Sanger sequencing revealed the inclusion of a 179-bp fragment of intron 2 in the *PpAN1* amplicon, resulting in a premature stop codon. The red box reflects the intronic sequence retained in the transcript. Asterisk, premature stop. F, Loss of purple pigmentation in CRISPR-edited *PgAN1* T_0_ plants. Left bar = 2 cm; right bar = 5 mm. G, CRISPR–Cas9-generated mutant alleles from the yellow T_0_ chimeric plants are shown. Dashed lines represent deletions. The bold letter indicates a single-nucleotide insertion.

We first sought to utilize our genomes to map the most conspicuous phenotype distinguishing the two species, nectar guide color variation*. Physalis grisea* displays deep purple nectar guides typical of most *Physalis* species, whereas *P. pruinosa* does not (Figure 2A). This pigmentation difference is also found on stems and branches. Crossing *P. grisea* and *P. pruinosa* resulted in F1 hybrids showing purple pigmentation, and an F2 population showed that the yellow color was segregated as a single recessive mutation. Mapping-by-sequencing localized the mutation to chromosome 4; however, limited recombination resulted in a large interval spanning most of the chromosome (Figure 2B).

To identify candidate genes, we searched for homologs of genes involved in the production of anthocyanins in the *Solanaceae* genus *Petunia*. Anthocyanins belong to a class of polyphenolic secondary metabolites named flavonoids, and one outcome of their accumulation in tissues and organs is purple pigmentation (Liu et al., 2018). Many ornamental *Petunia* species show variation in anthocyanin accumulation, and studies on this diversity have identified enzymes and transcription factors in the anthocyanin pathway (Bombarely et al., 2016; Liu et al., 2018).

Anthocyanin biosynthesis involves three major steps, including the conversion of phenylalanine to 4-coumaroyl-CoA through stepwise enzymatic reactions, and the conversion of 4-coumaroyl-CoA to dihydroflavonols, which are precursors in the final synthesis steps of specific anthocyanins (Figure 2C). We identified four orthologs of anthocyanin pathway genes and their regulators on chromosome 4. Overlaying our SV analysis revealed a mutation in only one of these genes, a 43-bp deletion in the second intron of the *P. pruinosa* gene Phypru04g010390, which encodes a basic Helix Loop Helix (bHLH) transcription factor ortholog of petunia ANTHOCYANIN1 (AN1) (Spelt et al., 2000; Figure 2D). AN1 activates the structural gene *DIHYDROFLAVONOL REDUCTASE* and other anthocyanin regulators (Spelt et al., 2000)*.* Notably, mutations in petunia *AN1* result in loss of anthocyanins in all tissues (Spelt et al., 2000, 2002). Using reverse transcription polymerase chain reaction (RT–PCR) and sequencing of cDNA, we found that *AN1* transcripts in *P. pruinosa* were longer than those in *P. grisea* due to a retention of 179 bp from intron 2, which results in a premature stop codon (Figure 2E). We validated this result by CRISPR–Cas9 targeting *PgAN1* (Phygri04g010290) in *P. grisea*. Five out of 11 first-generation (T_0_) transgenic lines failed to produce anthocyanins, and sequencing showed that these plants carried edited alleles of *PgAN1* (Figure 2, F and G). Though another variant closely linked to *AN1* on chromosome 4 could be responsible for the color variation, our genetic and molecular results strongly support that the SV in *P. pruinosa AN1* (*PprAN1*) underlies the absence of purple pigmentation in *P. pruinosa* and further demonstrate the utility of our genomic resources in deploying forward genetics in *Physalis.*

### The MADS-box genes *MPF2* and *MPF3* are not essential regulators of ICS

**Figure 3:**
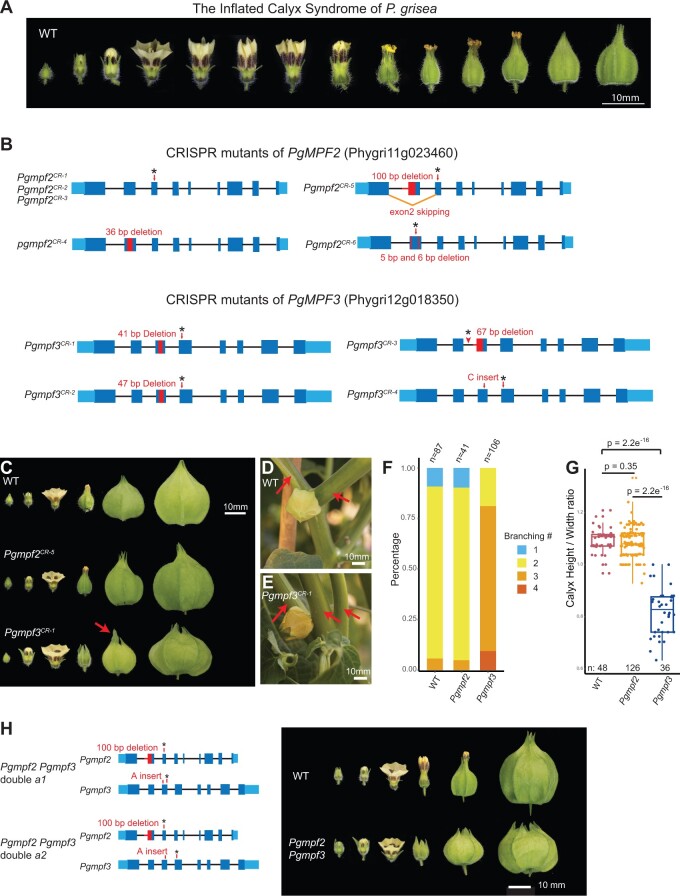
CRISPR–Cas9-generated mutants of the MADS-box genes *PgMPF2* and *PgMPF3* do not prevent ICS. A, Images showing sequential stages of ICS in *P. grisea* from early flower formation to calyx inflation over 3 days. Bar = 10 mm. B, Multiple, independently derived null alleles in *PgMPF2* and *PgMPF3*. Red boxes and lines, deletions; black asterisks, stop codons. Three alleles of *PgMPF2* (*Pgmpf2^CR-1^*, *Pgmpf2^CR-2^*, and *Pgmpf2^CR-3^*) with different mutations in exon 3 result in the same premature stop codon. Specific mutations for all alleles are shown in Supplemental Figure S2 and Supplemental Data Set 3. C–G, Phenotypes of *Pgmpf2* and *Pgmpf3* null mutants. All homozygous mutants independently derived alleles showed the same phenotypes, and *Pgmpf2^CR-5^* and *Pgmpf3^CR-1^* were used as references for phenotypic analyses. C, Calyx inflation is not disrupted in *Pgmpf2* and *Pgmpf3* mutants. Representative images from *Pgmpf2^CR-5^* and *Pgmpf3^CR-1^* are shown. The leaf-like sepal tip of *Pgmpf3^CR-1^* is indicated by the red arrow. Bar = 10 mm. D and E, Shoot branching phenotype of *Pgmpf3^CR-1^* compared to WT. A typical sympodial unit of WT *Physalis* consists of one leaf, one flower, and two side shoots. *Pgmpf3* mutants develop mostly three side shoots. Bar = 10 mm. E, Branches are indicated by red arrows in representative images. F, Quantification of branching in WT, *Pgmpf2*, and *Pgmpf3* shown as stacked bar charts. Branching counts are shown in Supplemental Data Set 4. G, Quantification of calyx height/width ratio in WT, *Pgmpf2*, and *Pgmpf3*. Raw measurements are shown in Supplemental Data Set 5. Statistical significance determined by two-tailed, two-sample *t* tests, and *P*-values are shown. H, Calyx inflation is not disrupted in *Pgmpf2 Pgmpf3* double mutants. Two allelic combinations in double mutants of *Pgmpf2 Pgmpf3* (*a1* and *a2*) displayed the same phenotype, and allele *a2* was used as a reference in the image shown. Bar = 10 mm.

The most striking feature of *Physalis* is the ICS, which evolved repeatedly in other *Solanaceae* genera and angiosperms (Paton, 1990; Padmaja et al., 2014; Deanna et al., 2019). Soon after fertilization, sepals undergo remarkable growth and expansion acropetally to encapsulate fruits in balloon-like papery husks, which may provide protection from pathogens and promote seed dispersal (Figure 3A; Baumann and Meier, 1993; Li et al., 2019). Despite long-standing interest, the evolutionary and mechanistic origins of ICS remain unclear. One early defining study proposed that heterotopic expression of *MPF2* was essential to the evolution of ICS (He and Saedler, 2005). This hypothesis was based on overexpression of the potato ortholog *StMADS16* in tobacco (*Nicotiana tabacum*), which produced leaf-like sepals. Empirical support in *Physalis* came from RNA interference (RNAi) knockdown of *MPF2* in *P. floridana*, where multiple transgenic lines showed a reduced calyx size, the severity of which was highly correlated with impaired fertility, but counterintuitively not the level of reduction of *MPF2* transcripts (He and Saedler, 2005).

Despite this contradictory result, follow-up studies proposed and tested an extended mechanism involving regulation of *MPF2* by the *AP1*-like transcription factor gene *MPF3* (ortholog of Arabidopsis *APETALA1* and tomato *MACROCALYX)*, in combination with hormonal control and fertilization (He and Saedler, 2007; Zhao et al., 2013). However, functional data supporting these conclusions were based on overexpression, plus also RNAi and virus-induced gene silencing (VIGS) knockdown of expression. Pleiotropic phenotypic outcomes are common in overexpression experiments, and are challenging to relate to specific genes studied, whereas RNAi and VIGS are difficult to interpret due to variable knockdown efficiencies and potential off-target effects (Xu et al., 2006; Senthil-Kumar and Mysore, 2011). Further convolution of a possible ICS mechanism emerged with the recent publication of the *P. floridana* genome, and the suggestion that absence of the *SEP4* ortholog of the tomato MADS-box gene *SlMBP21*/*J2* in *Physalis* was yet another critical factor in the origin of ICS (Lu et al., 2021).

To address these inconsistencies and provide a more robust genetic dissection of ICS, we first used CRISPR–Cas9 genome editing to eliminate *MPF2* and *MPF3* function in *P. grisea*. We generated five alleles of *PgMPF2* (Phygri11g023460) and four alleles of *PgMPF3* (Phygri12g018350) (Figure 3B), and these independent mutations caused different premature stop codons. Notably, none of these homozygous mutants disrupted ICS; all *Pgmpf2^CR^* mutants showed similar calyx inflation as wild-type (WT), and *Pgmpf3^CR^* mutants displayed enlarged and more leaf-like tips of sepals before inflation, a phenotype also observed in tomato *mc* mutants (Figure 3C; Yuste-Lisbona et al., 2016). Although this change of sepal tips was accompanied by a lower calyx height/width ratio (Figure 3G), inflation was unaffected. Besides the sepal phenotype, *Pgmpf3* also displayed abnormal branching patterns; *Pgmpf3* mutants frequently produced three instead of two sympodial shoots (Figure 3, D–F). Finally, we generated double mutants to test whether eliminating *PgMPF2* and *PgMPF3* functions together would disrupt inflation. Notably, *Pgmpf2 Pgmpf3* plants matched the phenotypes of *Pgmpf3* single mutants, including the progression of ICS (Figure 3H). In summary, these CRISPR–Cas9 engineered loss-of-function mutations in *PgMPF2* and *PgMPF3* show that these MADS-box genes are not responsible for the evolution of ICS and are not essential regulators of this developmental process.

#### Targeted mutagenesis of additional MADS-box genes does not abolish ICS

**Figure 4:**
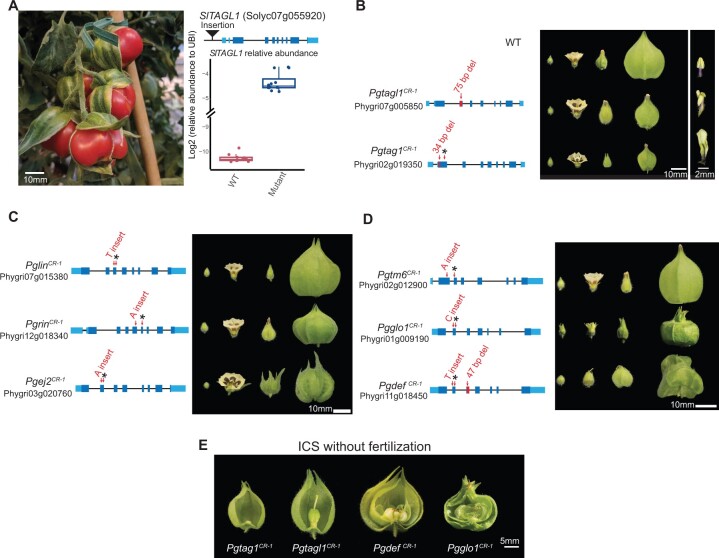
CRISPR–Cas9 generated mutations in eight additional candidate MADS-box genes do not disrupt ICS. A, Overexpression of *SlTAGL1* caused by a transposable element insertion (see “Materials and methods”) results in an enlarged calyx in tomato, mimicking ICS and presenting another candidate MADS-box gene. Left: image of calyx phenotype from the *SlTAGL1* mutant. Bar = 10 mm. Right, top: gene model of *SlTAGL1* with the transposon insertion (black triangle) identified by genome sequencing. Right, bottom: RT–qPCR on cDNA derived from young sepals showing overexpression of *SlTAGL1* in the mutant. Sepal tissue from three WT plants and four mutant plants was assayed (see “Materials and methods”); each data point represents one technical replicate. B, Mutations in *PgTAGL1* and *PgTAG1* cause homeotic transformations of stamens to petal-like organs but do not disrupt ICS. Middle image: representative calyx phenotypes at different developmental stages. Bar = 10 mm. Right image: single organs from the third floral whorl. Bar = 2 mm. C, Mutations in three *SEP4* homologs do not disrupt ICS. Bar = 10 mm. D, Mutations in multiple B-function MADS-box genes do not disrupt ICS. Bar = 10 mm. E, ICS still occurs in mutants with fertilization defects or those that fail to produce fruits. Mutations in *PgTAG1*, *PgTAGL1*, *PgDEF*, and *PgGlo1* cause homeotic transformations of floral organs that abolish self-fertilization, but ICS is preserved. Bar = 5 mm.

In an effort to identify genes involved in ICS, we embarked on a more comprehensive reverse genetics approach targeting MADS-box genes known to regulate floral organ development in tomato and other species, including additional MADS-box family members that mimic ICS when overexpressed or mutated in non-ICS *Solanaceae*. For example, we characterized a spontaneous tomato mutant with greatly enlarged fleshy fruit-covering sepals and found a transposon insertion SV upstream of *TOMATO AGAMOUS-LIKE1* (*TAGL1*) that caused more than 80-fold overexpression in developing sepals (Figure 4A). *TAGL1* belongs to the *AGAMOUS* clade of MADS-box transcription factors and is a close paralog of *TOMATO AGAMOUS 1 (TAG1).* Previous studies showed that both of these genes control flower development, and when either is overexpressed, enlarged and fleshy sepals are produced, in part mimicking ICS (Pnueli et al., 1994; Itkin et al., 2009). To test the roles of the *Physalis* orthologs of these genes, we generated CRISPR mutants. As observed in corresponding mutants of other species (Yanofsky et al., 1990; Pan et al., 2010), *Pgtag1^CR-1^* homozygous mutants displayed severe homeotic transformation of stamens to petal-like structures, while *Pgtagl1^CR-1^* displayed similar but weaker homeotic transformations (Figure 4B). Importantly, despite these floral organ defects, accompanied also by partial or complete loss of self-fertilization, both of these mutants maintained inflation, although calyx size was reduced, potentially due to secondary growth effects (Figure 4, B–E).

Based on their roles in floral organ development and inflorescence architecture, *SEPALLATA4 (SEP4*) MADS-box genes are another set of ICS candidates. Tomato has four *SEP4* clade MADS-box genes: *J2*, *SlMADS1/ENHANCER OF J2* (hereafter *EJ2*), *LONG INFLORESCENCE* (*LIN*), and *RIPENING INHIBITOR* (*RIN*)*.* We previously showed that *EJ2* and *LIN* regulate sepal development; mutants of *ej2* alone and in combination with *lin* develop enlarged sepals (Soyk et al., 2017). Analysis of the genome of *P. floridana* (Lu et al., 2021), and confirmed in our genomes, showed that *Physalis* lost the ortholog of *J2*, whereas the other three *SEP4* genes are present. Curiously, loss of *J2* was proposed to have promoted the evolution of ICS, but non-ICS *Solanaceae* such as pepper also lack *J2*. To test the roles of the *SEP4* clade in ICS, we used CRISPR–Cas9 to mutate all three *SEP4* genes in *P. grisea*. Notably, multiple independent mutations in *PgEJ2*, *PgLIN*, and *PgRIN* did not inhibit ICS. Similar to our findings in tomato *ej2* mutants (Soyk et al., 2017), mutants of *Pgej2^CR-1^* produced larger sepals in young and fully developed flowers, but inflation proceeded normally, with the only modification being sepal tips failing to coalesce to a single point after inflation is complete (Figure 4C).

#### Fertilization is not required for ICS

In flower development, B-class MADS-box genes participate in specifying petal and stamen identity, and the loss of B function leads to homeotic transformations of petals and stamens, which impaired self-fertilization (Yanofsky et al., 1990; Weigel and Meyerowitz, 1994; Theißen and Saedler, 2001). If fertilization-related signals were required for ICS, as reported (He and Saedler, 2007), mutations in B-class MADS-box genes should result in abnormal ICS development. Previously, a mutation deleting the B-class MADS-box gene *GLOBOSA1* (*GLO1)* was shown to develop a double-layered calyx phenotype in *P. floridana* when fertilized with WT pollen (Zhang et al., 2014). We identified four B-class MADS-box genes in *P. grisea*, including the four closest homologs of *GLO1*: *PgGLO1* (Phygri01g009190), *PgGLO2* (Phygri06g017940), *PgDEF* (Phygri11g018450), and *PgTM6* (Phygri02g012900). CRISPR–Cas9-induced null mutations in all four genes failed to disrupt ICS. Mutants of *Pgtm6^CR-1^* and *Pgglo2^CR-1^* appeared WT, whereas *Pgglo1^CR-1^* and *Pgdef^CR-1^* both displayed expected homeotic transformations of stamens to carpels and petals to sepals. Notably, calyx inflation was unaffected even in the second whorls of *Pgglo1^CR-1^* and *Pgdef^CR-1^* where petals were converted to sepals (Figure 4, D and E).

Fertility or signals from developing fruits have also been observed to be required for the initiation and progression of inflation, perhaps due to the activity and signaling of hormones such as cytokinin and gibberellin (He and Saedler, 2007). However, many of our MADS-box mutants with severe floral organ homeotic transformations also fail to self-fertilize and have various degrees of defects in fruit development. That ICS is unaffected in these mutants provides compelling genetic evidence that ICS can be uncoupled from normal fertilization. In particular, both *Pgdef ^CR-1^* and *Pgglo1^CR-1^* homozygous mutants cannot self-fertilize and form multiple small fruits without seeds due to homeotic transformations of stamens to carpels, yet the twin outer layers of sepals still form inflated calyces (Figure 4E). Moreover, in *Pgtagl1^CR-1^*and *Pgtag1^CR-1^*mutants, which cannot self-fertilize and whose fruits arrest early in development or fail to form entirely, respectively, inflation remained intact (Figure 4E).

In summary, although earlier observations, hypotheses, and data suggested critical roles of several MADS-box genes in the evolution of ICS, our results show that calyx inflation is maintained in loss-of-function mutants of the *P. grisea AG* clade, *SEP4* clade, and B-class MADS-box transcription factor genes. These data further demonstrate that although fertilization signals or developing fruit may contribute to the regulation of calyx inflation, neither is absolutely required.

### The *huskless* mutant, caused by a mutation in an *AP2*-like transcription factor, eliminates inflated calyx

**Figure 5:**
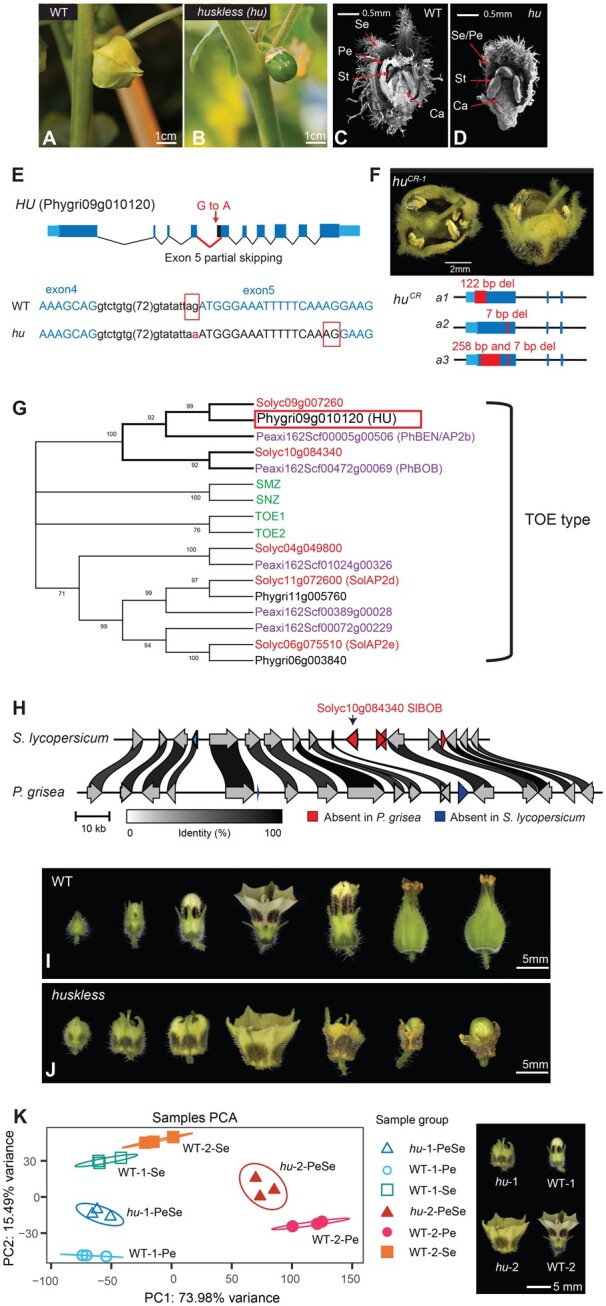
The *huskless* mutant lacks an inflated calyx due to mutation of an *AP2*-like transcription factor. A–D, Phenotypes of the EMS-derived *huskless* (*hu*) mutant. A and B, Images of WT and the *hu* mutant displaying the loss of calyx phenotype at the mature green fruit stage. Bar = 1 cm. C and D, Longitudinal SEM images of developing flowers of WT and *hu* showing *hu* mutants develop only three floral whorls compared to four in WT. The first whorl of *hu* flowers shows hallmarks of sepal and petal identity. Se: sepal; Pe: petal; St: stamen; Ca: Carpel. Bar = 0.5 mm. E, Gene model showing the G-to-A point mutation causing partial skipping of exon 5 in the *AP2-like* transcription factor gene *Phygri09g010120.* Blue-colored nucleotides represent exonic sequences; red boxes indicate 3’ splice sites in WT and *hu*. F, CRISPR–Cas9-generated mutations in *Phygri09g010120.* Top: gene models showing three independent CRISPR null alleles of *hu*. Sequences 3′ of the third intron are omitted. *hu^CR-1^* is homozygous for allele 1 (*a1*). Bottom: images of *hu^CR-1^* flower phenotype. Bar = 2 mm. G, Maximum likelihood consensus tree of the TOE-type euAP2 proteins from *A. thaliana* (gene names in green), *P. axillaris* (Peaxi IDs in purple), *S. lycopersicum* (Solyc IDs in red), and *P. grisea* (Phygri IDs in black). Bootstrap values (%) based on 500 replicates are indicated near the branching points; branches below 50% have collapsed. H, Local synteny analysis between *S. lycopersicum* and *P. grisea* showing the absence of the *Solyc10g084340* orthologue (petunia *BOB* orthologue) in *P. grisea*. Arrows indicate genes and orientations. Protein identity percentages between orthologues are indicated by ribbon shades in gray scale; only links above 80% identity are shown. I and J, Series of images of WT and *hu* developing flowers from before anthesis through early fruit development. Bar = 5 mm. K, PCA of WT and *hu* RNA-seq data. Right image: visual reference of the two stages used for expression profiling from WT and *hu* floral whorls. Numbers (−1 or −2) in the sample groups represent stage 1 or 2; petal or sepal whorls in WT are denoted as Pe, Se respectively; PeSe represents the merged outer whorl in *hu*. The top 3,000 DEGs were used for PCA. Bar = 5 mm.

Forward genetics is a powerful and unbiased approach to identify genes controlling traits of interest in model systems. We performed a small-scale ethyl methanesulfonate (EMS) mutagenesis screen in *P. grisea* to identify genes involved in calyx development (see “Materials and methods”). A recessive mutant bearing fruits without husks was identified and named *huskless* (*hu*) (Figure 5, A and B). Scanning electron microscope (SEM) imaging of dissected flower buds showed that *hu* mutants developed three floral whorls instead of four compared to WT (Figure 5, C and D). To isolate the causative mutation, we sequenced genomic DNA from a pool of *hu* mutants and WT siblings from the original *P. grisea* mutagenesis (M2) family (see “Materials and methods”). Aligning Illumina-sequenced reads to the *P. grisea* genome allowed screening for single-nucleotide variants (SNVs) that were homozygous in the *hu* pool but not in the WT sibling pool. We scored these SNVs for predicted functional consequences on annotated gene transcripts using SnpEff (Cingolani et al., 2012). Out of eight such SNVs, one was a G-to-A mutation in a 3′-splice site of Phygri09g010120, which encodes an APETALA2 (AP2)-like transcription factor (Figure 5E; Supplemental Table S6). Co-segregation analysis in M3 families confirmed the association of this mutation with the *hu* phenotype (Supplemental Table S7), and sequencing RT–PCR products of Phygri09g010120 from *hu* floral tissue showed mis-splicing in the fourth intron, resulting in partial skipping of exon 5 (Figure 5E). Importantly, independent CRISPR-generated mutations of this *AP2-like* gene in *P. grisea* resulted in independent mutations that caused the same phenotype as *hu* (Figure 5F).

*HU* is the homolog of *P. hybrida AP2B/BLIND ENHANCER* (*BEN*) (Figure 5G), which specifies the second and third floral whorl identity (Morel et al., 2017) with its redundant paralog *BROTHER OF BEN* (*BOB*). Petal development is strongly inhibited in *ben bob* double mutants, resulting in severely reduced or absent petals, and partial conversion of sepals into petals, resembling *hu* (Morel et al., 2017). Because the *P. hybrida* genome is highly fragmented (Bombarely et al., 2016), we performed a synteny analysis of the chromosomal segments containing *BOB* in *P. grisea, P. pruinosa*, and *S. lycopersicum* and found that this paralog of *HU* (*BEN*) is present in tomato but not in groundcherry (Figure 5H). Thus, *hu* emerged in our forward genetics mutagenesis screen, because the *BOB* ortholog and therefore redundancy is absent in *P. grisea*.

The first floral whorl of *hu* displays characteristics of both sepals and petals (Figure 5, I and J). The whorl begins developing with green as the dominant color, like sepals, but gradually turns yellow as the flower matures, maintaining green color at organ tips. Nectar guides are also visible throughout the development of the first whorl, indicative of early petal identity. After fertilization, the first whorl mildly increases in size but fails to fully inflate before gradually senescing as *hu* fruits develop into the size of WT fruits.

To characterize the role of *HU* in whorl identity and ICS, we profiled transcriptomes by RNA-seq from WT sepals and petals at two stages of organ maturation and compared them with corresponding stages of *hu* first whorls (Figure 5K; see “Materials and methods”). Principal component analysis (PCA) revealed *hu* expression profiles (denoted as *hu*-PeSe) were positioned between the profiles of WT sepals and petals at both stages, supporting the mixed-organ identity observed phenotypically. Thus, the loss of the inflated calyx in *hu* mutants is from a failure to properly specify sepal and petal identity as opposed to directly disrupting a mechanistic origin of ICS. Our identification of *hu* through forward genetics exemplifies how the presence–absence variation of paralogs can shape genetic redundancies and genotype-to-phenotype relationships in related lineages, and further illustrates the value of multiple related model systems.

## Discussion

Discoveries in plant development, cell biology, and genetics continue to depend on a limited number of model systems, often centered around *A. thaliana* and its relatives in the Brassicaceae family (Chang et al., 2016). New models are essential to advance fundamental and applied research beyond the small amount of biological diversity captured by current models. While additional model species have been proposed or are under development (Chang et al., 2016), most lack the powerful combination of efficient genomics and genetics. Moreover, emphasis is largely on neglected lineages and single representative species within them. An approach with complementary benefits relies on multiple models within a lineage to address often overlooked questions of species-specific and comparative evolutionary history over short time frames. The *Solanaceae* family is ideal in this regard, including: (1) rich diversity throughout approximately 100 genera and ˃3,000 species spanning ∼30 million years of evolution; (2) broad agricultural importance from ˃2 dozen major and minor fruit and vegetable crops; and (3) feasibility of rapidly developing and integrating genome editing with reference and pangenome resources.

By establishing high-quality chromosome-scale assemblies for *P. grisea* and *P. pruinosa*, we developed these *Physalis* species as new models to advance *Solanaceae* systems with genomics and genetics. Most significantly, our integration of these resources revealed that the mechanisms underlying ICS remain elusive. Indeed, despite previous evidence suggesting otherwise, we conclude that none of the 11 candidate MADS-box genes we functionally characterized using genome editing, nor fertility alone, are core regulators of ICS. Our findings therefore force a reset in the search for the physiological, genetic, and molecular mechanistic origins of this evolutionary novelty. Though a logical starting point, the candidate gene approach based on MADS-box overexpression phenotypes in other species was prone to misleading hypotheses and false positives, likely due to the complex evolutionary history of the MADS-box family members and their even more complex genetic and physical interactions. Indeed, multiple MADS-box genes appear to be capable of mimicking ICS through overexpression, possibly due to coordinated activation of closely related paralogs and subsequent complex feedback regulation and interactions among other family members. This might suggest double and higher-order mutants of these or other MADS-box genes not investigated here would ultimately perturb ICS, possibly reflecting a collective role of multiple family members acting redundantly or in a network. However, such a result would not necessarily indicate direct roles for these genes in the evolutionary steps leading to ICS.

Based on our genetics, we expect additional or other genes and molecular programs to be central, and the tools established here provide the foundation to revisit ICS in an unbiased way. ICS is a rapid and dynamic process, where extraordinary morphological changes in sepal growth and inflation occur within a few days. This suggests that the molecular events driving and responding to the inception of the transition from a noninflated sepal whorl to active inflation may be short-lived, happening in the order of hours. We propose that the future dissection of ICS should be based on detailed and integrated temporal, morphological, and molecular analyses to capture these transient events. A recent study in tomato took advantage of transcriptome profiling and computational ordering of hundreds of single-shoot apical meristems to capture and reconstruct a highly detailed temporal gene expression map of the floral transition. These data revealed previously hidden genes, short-lived expression programs, and several genes that function in parallel transient pathways critical to the floral transition process (Meir et al., 2021). With the new reference genomes and annotations of *P. grisea* and *P. pruinosa*, a similar approach can be applied to ICS, where large numbers of individual sepals can readily and reliably be harvested and profiled throughout calyx development. As opposed to focusing on entire floral buds (Gao et al., 2020), such high-resolution temporal transcriptome profiling of sepals alone would provide comprehensive and unbiased information regarding global and possibly gene-specific molecular signatures in the initiation and maintenance of inflation, and expose new candidates that can be studied using the integrated genomics and genome editing strategies demonstrated here.

Beyond floral development and ICS in *Physalis*, our work sets a high-quality anchor to broaden biological questions and discoveries in the *Solanaceae*, and further illustrates fast and efficient approaches to building new model systems. Establishing new pangenome and genome editing tools in many additional genera of *Solanaceae* and of other plant families will enable comparative genomic and genetic studies over both short and long evolutionary timescales.

## Materials and methods

### Plant material, growth conditions, and phenotyping

Seeds of *P. grisea* and *P. pruinosa* were obtained from the Solanaceae Germplasm Bank at the Botanical Garden of Nijmegen and from commercial seed sources. Seeds were directly sown into the soil (PRO-MIX BX Mycorrhizae Growing Mix) in 96-well plastic flats and grown in the greenhouse under long-day conditions (16-h light/8-h dark) supplemented with artificial light from high-pressure sodium bulbs (∼250 μmol m^−2^ s^−1^). The temperature ranged from 26°C to 28°C during the day to 18°C–20°C during the night, with a relative humidity of 40%–60%. Four-week-old seedlings were transplanted to 4-L pots filled with soil (PRO-MIX HP Mycorrhizae Growing Mix) in the same greenhouse, or into the fields at Cold Spring Harbor Laboratory unless otherwise noted. The tomato mutant displaying enlarged fleshy sepals from Figure 4 was a gift from Dr. Dani Zamir, which arose from the whole-genome backcross lines constructed from a cross between *Solanum pimpinellifolium* (LA1589) and *S. lycopersicum* inbred variety cv. E6203 (TA209) (Grandillo and Tanksley, 1996). Branching and internode length phenotypes were assayed in greenhouse-grown plants 2 months after sowing.

### Extraction of high-molecular weight DNA and long-read sequencing

For long-read sequencing, shoot apices of 3-week-old seedlings were harvested after a 48-h dark treatment. Extraction of high-molecular weight genomic DNA, construction of ONT libraries and PacBio HiFi libraries, and sequencing were described previously (Alonge et al., 2020, 2021). Hi-C experiments were conducted at Arima Genomics (San Diego, CA, USA) from 2 g of flash-frozen leaf tissue.

### *Physalis grisea* chloroplast and mitochondria genome assembly

To assemble the *P. grisea* chloroplast genome, all HiFi reads were aligned to the previously published *Physalis* chloroplast reference genome (GenBank ID MH019243.1) with Minimap2 (v2.17-r974-dirty, -k19 -w19) (Li, 2018). All reads with at least one primary alignment spanning at least 90% of the read were assembled with HiCanu (version 2.0, genomeSize = 155k) (Nurk et al., 2020). The three resulting HiCanu unitigs were aligned to themselves with Nucmer (v3.1, –maxmatch) (Kurtz et al., 2004) and manually joined to produce a single trimmed and circularized contig. The contig was rotated to start at the same position as the reference. Liftoff was used to annotate the *P. grisea* chloroplast genome (Shumate and Salzberg, 2021).

*Physalis grisea* mitochondrial contigs were extracted from the polished ONT Flye assembly (see below). To identify mitochondrial contigs, tobacco (*N. tabacum*), pepper (*C. annuum*), tomato (*S. lycopersicum*), and eggplant (*S. melongena*) mitochondrial transcript sequences (GenBank IDs NC_006581.1, NC_024624.1, NC_035963.1, and NC_050334.1, respectively) (Sugiyama et al., 2005) were extracted with gffread (Pertea and Pertea, 2020) and aligned to the ONT Flye assembly with Minimap2 (v2.17-r941, -x splice). For each query transcriptome, any ONT contig shorter than 500 kb with at least one alignment at least 100-bp long was considered, and any such contig identified by at least two query transcriptomes was labeled as mitochondrial. These contigs were aligned to the *P. grisea* chloroplast genome which indicated that they were all mitochondrial and not chloroplast sequences. These ONT mitochondrial sequences were aligned to the raw HiCanu contigs (see below) with Nucmer (v3.1, –maxmatch), and nine ONT contigs were manually replaced with two homologous HiCanu contigs. Liftoff was used to annotate the *P. grisea* mitochondrial genome using the *S. melongena* annotation as evidence.

### *P. grisea* genome assembly

*Physalis grisea* HiFi reads were assembled with HiCanu (version 2.0, genomeSize = 1,500 m). *Physalis grisea* ONT reads at least 38 kbp long and with an average quality score of at least Q12 were assembled with Flye (v2.8.1-b1676, –genome-size 1.5g) (Kolmogorov et al., 2019). The Flye contigs were iteratively polished for two rounds with Freebayes (Garrison and Marth, 2012). About 200,000,000 Illumina short reads (SRA ID SRR7066586) were randomly sampled with seqtk (https://github.com/lh3/seqtk) and aligned to the Flye contigs with BWA-MEM (v0.7.17-r1198-dirty) (Li, 2013). Alignments were sorted and indexed with samtools (Patro et al., 2017). Freebayes was used to call variants (v1.3.2-dirty, –skip-coverage 480) and polishing edits were incorporated with bcftools consensus (-i'QUAL > 1 && (GT = "AA" ‖ GT = "Aa")' -Hla) (Danecek et al., 2021).

The HiCanu contigs were aligned to the *P. grisea* chloroplast and mitochondria genomes with minimap2 (v2.17-r941, -x asm5), and any contigs covered ˃50% by alignments were removed. Potential bacterial contaminant sequences were screened out using a process similar to that used by the Vertebrate Genomes Project (Rhie et al., 2021). The HiCanu contigs were first masked with windowmasker (version 1.0.0, -mk_counts -sformat obinary -genome_size 1448242897) (Morgulis et al., 2006). Then, the HiCanu contigs were aligned to all RefSeq bacterial genomes (downloaded on May 21, 2020) with BLAST (version 2.5.0, -task megablast -outfmt "6 std score" -window_masker_db) (Altschul et al., 1990). Any contigs with at least one alignment with an E-value ˂0.0001, a score of at least 100, and a percent-identity of at least 98% were manually inspected, and one contig was removed. To remove potential false haplotypic duplication, HiFi reads were aligned to the screened contigs with Minimap2 (v2.17-r941, -x asm5), and any contigs with at least 50% of the contig with less than 5× coverage were purged (Guan et al., 2020)

The screened and purged contigs were patched with Grafter (https://github.com/mkirsche/Grafter), a beta version of RagTag “patch” (Alonge et al., 2021). Polished ONT contigs were aligned to the HiCanu contigs with Nucmer (v3.1, -maxmatch -l 100 -c 500) and these alignments were used by Grafter to make patches (minq = 0 min_weight_supp = 10 min_weight = 10). Patched contigs were then scaffolded with Bionano optical maps generated at the McDonnell Genome Institute at Washington University. Finally, chromosome-scale scaffolds were manually derived with Hi-C using Juicebox Assembly Tools (Dudchenko et al., 2018). To identify and correct potential misassemblies, HiFi and ONT reads were aligned to the scaffolds with Winnowmap (v1.11, -ax map-pb, and -ax map-ont, respectively) and SVs were called with Sniffles (v1.0.12, -d 50 -n -1 -s 3) (Jain et al., 2020b). We removed any SVs with ˂30% of reads supporting the alternative allele and we merged the filtered SV calls with Jasmine (version 1.0.10, max_dist = 500 spec_reads = 3 –output_genotypes) (Kirsche et al., 2021). After merging and manually inspecting the SV calls, a total of four misassemblies were manually corrected. VecScreen did not identify any “strong” or “moderate” hits to the adaptor contamination database (ftp://ftp.ncbi.nlm.nih.gov/pub/kitts/adaptors_for_screening_euks.fa) (https://www.ncbi.nlm.nih.gov/tools/vecscreen/). Finally, we removed any unplaced contigs shorter than 1 kbp. Mercury was used to compute QV and completeness metrics (k = 21) (Rhie et al., 2020).

### *P. pruinosa* genome assembly

The *P. pruinosa* genome was assembled just as the *P. grisea* genome, with the following distinctions. HiFi reads were assembled with Hifiasm instead of HiCanu (v0.13-r308, -l0) (Cheng et al., 2021). Also, neither a chloroplast nor a mitochondria genome was assembled for *P. pruinosa*. To screen organellar contigs, raw Hifiasm primary contigs were aligned to the *P. pruinosa* reference chloroplast genome (GenBank ID MH019243.1) and the *P. grisea* mitochondria genome. As with *P. grisea*, SVs were called to identify potential misassemblies, and no misassemblies were found in the *P. pruinosa* scaffolds.

### Gene and repeat annotation

Raw RNASeq reads from *P. grisea* were assessed for quality using FastQC version 0.11.9 (Andrews, 2010), and were then aligned to the *P. grisea* assembly using STAR aligner (Dobin et al., 2013). Finally, reference-based transcripts were assembled using StringTie v2.1.2 (Pertea et al., 2015). We used the portcullis v1.2.0 (Mapleson et al., 2018) method to filter out the invalid splice junctions from the bam alignments. Additionally, we lifted orthologs from the Heinz ITAG4.0 annotation (Hosmani et al., 2019) and the pangenome annotation (Gao et al., 2019) using the Liftoff v1.6.1(-exclude_partial -copies) (Shumate and Salzberg, 2021) pipeline. Structural gene annotations were then generated using the Mikado v2.0rc2 (Venturini et al., 2018) framework using the evidence set mentioned above following the Snakemake-based pipeline [Daijin]. Functional annotation of the Mikado gene models was identified using the blastp alignments to uniprot/swissprot (Bairoch and Apweiler, 2000), TREMBL, Heinz ITAG4.0, and pan genome proteins database (Gao et al., 2019; Hosmani et al., 2019) and transferred using the AHRD pipeline (https://github.com/asishallab/AHRD). The *P. pruinosa* assembly was gene-annotated with Liftoff, using the *P. grisea* gene annotation as evidence (-copies). Transposable elements were annotated with EDTA (v1.9.6, –sensitive 1 –anno 1 –evaluate 1 –cds) (Ou et al., 2019). BUSCO was run on each genome assembly using the “embryophyta_odb10” lineage database (v5.0.0, -e 1e-05 –augustus –long) (Simão et al., 2015).

### SV detection

Structural variation between *P. grisea* and *P. pruinosa* was identified using the same pipeline used to identify SV-like misassemblies described above. However, instead of aligning *P. grisea* reads to the *P. grisea* assembly and *P. pruinosa* reads to the *P. pruinosa* assembly, *P. grisea* reads were aligned to the *P. pruinosa* assembly and *P. pruinosa* reads were aligned to the *P. grisea* assembly. Also, Winnowmap2 (version 2.0) was used instead of Winnowmap for alignments (Jain et al., 2020a). SVs intersecting genomic features in Figure 1G were counted as previously described (Alonge et al., 2020) based on *P. grisea* annotation version 1.3.0.

### CRISPR–Cas9 mutagenesis, plant transformation, and selection of mutant alleles

CRISPR–Cas9 mutagenesis was performed following our protocol as previously described (Lemmon et al., 2018; Swartwood and van Eck, 2019). Gene IDs related to this study are listed in Supplemental Table S8. Briefly, guide RNAs (gRNAs) were designed to be used in the Golden Gate cloning system (all gRNAs used in this study are listed in Supplemental Table S9 and were assembled into Level 1 (L1) constructs under the control of the U6 promoter. L1 guide constructs were then assembled with Level 1 constructs pICH47732-*NOS_pro_:NPTII* and pICH47742-*35S_pro_:Cas9* into the binary Level 2 vector pAGM4723. The final binary vectors were then transformed into groundcherry by *Agrobacterium tumefaciens*-mediated transformation through tissue culture (Swartwood and van Eck, 2019). Multiple independent first-generation transgenic plants (T_0_) were genotyped with specific primers surrounding the target sites. T_0_ plants were self-pollinated and the T_1_ generation was genotyped for the target genes and the presence or absence of the CRISPR–Cas9 transgene. We noticed that tissue culture and transformation resulted in a variable frequency of tetraploidy. All mutants were verified as homozygous or biallelic and having only mutant alleles.

### Tissue collection, RNA extraction, RT–PCR, and RT–qPCR

All tissues used were immediately frozen in liquid nitrogen before RNA extraction. For the analysis of *AN1* transcripts in *P. grisea* and *P. pruinosa*, young flower buds were harvested. For *TAGL1* gene expression analysis in the tomato calyx mutant, developing sepals at the open flower stage were harvested. Sepal tissue from three different WT plants and four different mutant plants was assayed as three biological replicates and four biological replicates, respectively. For the analysis of *huskless* (*hu*) and WT sepal gene expression profiles, the first whorl of *hu*, and WT sepals and petals at the stages shown in Figure 5K were harvested. Total RNA was extracted with the Zymo Research Quick-RNA Microprep kit following the manufacturer’s protocol. cDNA synthesis was performed using SuperScript IV VILO Master Mix (Thermo Fisher Scientific, Waltham, MA, USA) with 500 ng to 1,500 ng total RNA input. RT**–**PCR was performed with KOD One PCR Master Mix and primers listed in Supplemental Table S10. RT**–**qPCR was performed using Fast SYBR Green Master Mix with primers listed in Supplemental Table S10 on the Applied Biosystems QuantStudio version 6 system.

### Transcriptome analysis of *huskless* and WT

RNA-seq and differentially expressed genes (DEGs) analyses were performed as previously described with slight modification (Kwon et al., 2022). Briefly, the libraries for RNA-seq were prepared by the KAPA mRNA HyperPrep Kit (Roche, Basel, Switzerland). Paired-end 150-base sequencing was conducted on the Illumina sequencing platform (NextSeq, High-Output). Reads for WT and *hu* were trimmed by quality using Trimmomatic (Bolger et al., 2014) (version 0.39, parameters: ILLUMINACLIP:TruSeq3-PE-2.fa:2:40:15:1:FALSE LEADING:30 TRAILING:30 MINLEN:50) and quantified to the reference transcriptome assembly of *P. grisea* version 1.3.2 using Salmon version 1.4.0 (Patro et al., 2017). Quantification results from Salmon were imported into R using tximport version 1.24.0 (Soneson et al., 2016). PCA analysis of samples was performed and plotted using DEseq2 version 1.36.0 (Love et al., 2014) and pcaExplorer version 2.22.0 (Marini and Binder, 2019) with counts of the top 3,000 variable genes.

### Mapping of the yellow nectar guide variant

The yellow-guide trait displayed classical patterns of Mendelian inheritance of a single recessive gene in the F1 and F2 populations from the cross between *P. grisea* and *P. pruinosa*. A bulk segregant analysis was performed using 20 plants from each of the yellow-guide pool and purple-guide pool in the F2 segregating population. All reads were assessed for overall quality by FastQC version 0.11.9 (Andrews, 2010). Read mapping, variant calling, and SNP-index calculation of the Illumina reads from each pool were done by QTL-seq version 2.2.2 (Takagi et al., 2013). Parameters used for the sliding window SNP-index calculation by the qtlplot command were -n1 20 -n2 20 -F 2 -D 250 -d 5 -w 1000 -s 50. The calculated SNP index in each sliding window was imported into R (R Core Team, 2020) for the final plot.

### EMS mutagenesis and mutant screening in *P. grisea*

A small-scale EMS mutagenesis was performed using ∼1,500 *P. grisea* seeds (measured by weight). Seeds were soaked in distilled water overnight and then treated with 0.2% EMS (Sigma Aldrich, St. Louis, MO, USA) for 6 h. After treatment, seeds were washed with distilled water thoroughly and sowed into 96-well flats. Four-week-old seedlings were then transplanted into the field. When harvesting, fruits from every four M_1_ plants were bulk harvested into one group. For mutant screening, 80 groups of M2s were sowed, transplanted, and screened for sepal-related phenotypes.

### Mapping of *huskless*

Three *huskless* phenotype plants were identified from the same group. The pooled DNA from the three mutants, and the pooled DNA from 30 WT-looking siblings from the same group, were obtained by CTAB extraction methods. Libraries were prepared for sequencing using the Kapa Hyper PCR-free Kit and sequenced on Illumina Nextseq (PE150, high output). All reads were assessed for overall quality by FastQC version 0.11.9 (Andrews, 2010), and trimmed with Trimmomatic version 0.39 (Bolger et al., 2014) with parameters ILLUMINACLIP:TruSeq3-PE.fa:2:40:15:1:FALSE LEADING:30 TRAILING:30 MINLEN:75 TOPHRED33. Trimmed paired reads were mapped to the reference *P. grisea* genome using BWA-MEM (Li, 2013). Alignments were then sorted with samtools (Li et al., 2009). and duplicates marked with PicardTools (Picard Toolkit, 2019). Variants were called with freebayes (Garrison and Marth, 2012) and filtered with VCFtools (Danecek et al., 2011) for SNPs with a minimum read depth of 3 and minimum quality value of 20. SNPs that are homozygous in the mutant pool but not homozygous in the WT sibling pool were analyzed for effects on transcripts with snpEff (Cingolani et al., 2012) with *P. grisea* annotation version 1.3.0.

### Molecular phylogenetic analyses

In order to determine the phylogenetic relationship between the eleven selected *Solanaceae* species, 18 genomes were used to define orthogroups by Conservatory (Hendelman et al., 2021). Protein sequences of the twenty most conserved orthogroups genes were aligned with MAFFT (version 7.487) FFT-NS-2 (Katoh and Standley, 2013) (see Supplemental Data Set 6), before constructing the tree by IQ-tree with the following parameters -st AA -b 100 -pers 0.5 –wbtl (Minh et al., 2020). For the phylogenetic analysis of AP2-like proteins, protein sequences of the orthologs were retrieved from *P. grisea*, *S. lycopersicum*, and *P. axillaris* by BLAST (Altschul et al., 1990). Protein sequences (see Supplemental Data Set 7) were imported in MEGA version 11 (Tamura et al., 2021) and aligned with MUSCLE (default parameters). The tree was constructed using the maximum likelihood method and JTT matrix-based model. Bootstrap values (%) based on 500 replicates are indicated near the branching points; branches ˂50% have been collapsed. Alignment and tree files are provided as Supplemental Files S1 and S2.

### Synteny analysis at the *SlBOB* locus

Because the scaffold quality of the *P. axillaris* genome in the vicinity of *BOB* was suboptimal, we used SL4.0 with the *P. grisea* genome for the analysis. A BLAST search using Petunia *BOB* and *SlBOB* cDNA query sequences against the *P. grisea* genome failed to retrieve a high-confidence hit other than Phygri09g010120, which is the *BEN* ortholog. BLAST search of genes upstream and downstream of *SlBOB* located their syntenic regions in the *P. grisea* genome. Genomic sequences with annotations from Solyc10g084240 to Solyc10g084420, and from Phygri10g011780 toPhygri10g011960 were used in clinker version 0.0.23 (Gilchrist and Chooi, 2021) to generate gene translation alignments and visualizations.

### Accession numbers

Genome assemblies and annotations are available at https://github.com/pan-sol/pan-sol-data/tree/main/Physalis. Raw sequence data from this article can be found in Sequence Read Archive (SRA) under the BioProject PRJNA862958.

## Supplemental data

The following materials are available in the online version of this article.

**Supplemental Figure S1.** Hi-C heatmaps confirm reference assembly structural accuracy.

**Supplemental Figure S2.** Illustrations of CRISPR-engineered mutations in this study.

**Supplemental Figure S3.** Maximum likelihood consensus tree of the euAP2 proteins from *A. thaliana*, *P. axillaris*, *S. lycopersicum*, and *P. grisea*.

**Supplemental Table S1.** Genome assembly statistics.

**Supplemental Table S2.** Annotation stats of *P. grisea* and *P. pruinosa* genomes.

**Supplemental Table S3.** Result summary of SNP calls of *P. pruinosa* Illumina reads against *P. grisea* as reference.

**Supplemental Table S4.** High-impact SNP calls of *P. pruinosa* Illumina reads against *P. grisea* as reference.

**Supplemental Table S5.** SVs intersecting CDS.

**Supplemental Table S6.** SNPs with predicted high impact on transcripts of *huskless*.

**Supplemental Table S7.** Co-segregation test of the G/A SNP in Phygri09g010120 and the huskless phenotype.

**Supplemental Table S8.** Genes related to work in this study.

**Supplemental Table S9.** CRISPR guides used in this study.

**Supplemental Table S10.** Primers used in this study.

**Supplemental Data Set 1.** Internode length measurement of *P. grisea* and *P. pruinosa* related to Figure 1, B and C.

**Supplemental Data Set 2.** SVs intersecting genes.

**Supplemental Data Set 3.** CRISPR-generated mutations in this study.

**Supplemental Data Set 4.** Branching phenotype counts for WT, *Pgmpf2*, and *Pgmpf3* related to Figure 3F.

**Supplemental Data Set 5.** Calyx length and width measurement of WT, *Pgmpf2*, and *Pgmpf3* related to Figure 3G.

**Supplemental Data Set 6.** Protein sequences used for the phylogenic analysis of Solanaceae species in Figure 1A.

**Supplemental Data Set 7.** Protein sequences used for the phylogenetic analysis of AP2-like proteins in Figure 5G.

**Supplemental Data Set 8.** Statistical analysis tables.

**Supplemental File S1.** Tree file for the phylogenetic analysis in Figure 1A.

**Supplemental File S2.** Tree file for the phylogenetic analyses in Figure 5G and Supplemental Figure S3.

## Supplementary Material

koac305_Supplementary_Data

## Notes

M.C.S. and Z.B.L. conceived, designed, and led the study, and analyzed the data. J.H. led and coordinated the experiments and analyses. M.C.S. and Z.B.L. performed the genome sequencing, M.A. and M.C.S. generated the genome assemblies. S.R. annotated the genomes. M.B. and S.S. prepared DNA for long-read sequencing. S.S. performed the genome sequencing and analysis to identify the tomato ICS mimic mutation. N.T.R. contributed the CRISPR construct targeting *PgMPF3*. J.V.E. led the CRISPR transformations and generated all the CRISPR T_0_ lines. A.H. contributed to the phylogenetic analyses. J.H., M.A., and Z.B.L. prepared the figures and wrote the manuscript. All authors read, edited, and approved the manuscript.

The author responsible for distribution of materials integral to the findings presented in this article in accordance with the policy described in the Instructions for Authors (https://academic.oup.com/plcell) is: Zachary B. Lippman (lippman@cshl.edu).

## References

[koac305-B1] Alonge M , Lebeigle L, Kirsche M, Aganezov S, Wang X, Lippman ZB, Schatz MC, Soyk S (2021) Automated assembly scaffolding elevates a new tomato system for high-throughput genome editing. BioRxiv, 10.1101/2021.11.18.469135 PMC9753292 36522651

[koac305-B2] Alonge M , Wang X, Benoit M, Soyk S, Pereira L, Zhang L, Suresh H, Ramakrishnan S, Maumus F, Ciren D, et al (2020) Major impacts of widespread structural variation on gene expression and crop improvement in tomato. Cell 182: 145–161.e23 32553272 10.1016/j.cell.2020.05.021 PMC7354227

[koac305-B3] Altschul SF , Gish W, Miller W, Myers EW, Lipman DJ (1990) Basic local alignment search tool. J Mol Biol 215: 403–410 2231712 10.1016/S0022-2836(05)80360-2

[koac305-B4] Añibarro-Ortega M , Pinela J, Alexopoulos A, Petropoulos SA, Ferreira ICFR, Barros L (2022) Chapter Four - The powerful Solanaceae: food and nutraceutical applications in a sustainable world. *In* Toldrá F, ed, Advances in Food and Nutrition Research, Vol. 100. Academic Press, Cambridge, MA, pp 131–172 10.1016/bs.afnr.2022.03.004 35659351

[koac305-B18] Andrews S (2010) FastQC: A Quality Control Tool for High Throughput Sequence Data [Online]. http://www.bioinformatics.babraham.ac.uk/projects/fastqc/

[koac305-B5] Bairoch A , Apweiler R (2000) The SWISS-PROT protein sequence database and its supplement TrEMBL in 2000. Nucleic Acids Res 28: 45–48 10592178 10.1093/nar/28.1.45 PMC102476

[koac305-B6] Baumann TW , Meier CM (1993) Chemical defence by withanolides during fruit development in *Physalis peruviana*. Phytochemistry 33: 317–321

[koac305-B7] Bolger AM , Lohse M, Usadel B (2014) Trimmomatic: a flexible trimmer for Illumina sequence data. Bioinformatics 30: 2114–2120 24695404 10.1093/bioinformatics/btu170 PMC4103590

[koac305-B8] Bombarely A , Moser M, Amrad A, Bao M, Bapaume L, Barry CS, Bliek M, Boersma MR, Borghi L, Bruggmann R, et al (2016) Insight into the evolution of the Solanaceae from the parental genomes of *Petunia hybrida*. Nat Plants 2: 16074 27255838 10.1038/nplants.2016.74

[koac305-B9] Chang C , Bowman JL, Meyerowitz EM (2016) Field guide to plant model systems. Cell 167: 325–339 27716506 10.1016/j.cell.2016.08.031 PMC5068971

[koac305-B10] Cheng H , Concepcion GT, Feng X, Zhang H, Li H (2021) Haplotype-resolved de novo assembly using phased assembly graphs with hifiasm. Nat Methods 18: 170–175 33526886 10.1038/s41592-020-01056-5 PMC7961889

[koac305-B11] Cingolani P , Platts A, Wang LL, Coon M, Nguyen T, Wang L, Land SJ, Lu X, Ruden DM (2012) A program for annotating and predicting the effects of single nucleotide polymorphisms, SnpEff Fly 6: 80–92 22728672 10.4161/fly.19695 PMC3679285

[koac305-B12] Danecek P , Auton A, Abecasis G, Albers CA, Banks E, DePristo MA, Handsaker RE, Lunter G, Marth GT, Sherry ST, et al (2011) The variant call format and VCFtools. Bioinformatics 27: 2156–2158 21653522 10.1093/bioinformatics/btr330 PMC3137218

[koac305-B13] Danecek P , Bonfield JK, Liddle J, Marshall J, Ohan V, Pollard MO, Whitwham A, Keane T, McCarthy SA, Davies RM, et al (2021) Twelve years of SAMtools and BCFtools. GigaScience 10: giab008 33590861 10.1093/gigascience/giab008 PMC7931819

[koac305-B14] Deanna R , Larter MD, Barboza GE, Smith SD (2019) Repeated evolution of a morphological novelty: a phylogenetic analysis of the inflated fruiting calyx in the Physalideae tribe (Solanaceae). Am J Bot 106: 270–279 30779447 10.1002/ajb2.1242

[koac305-B15] Deanna R , Wilf P, Gandolfo MA (2020) New physaloid fruit-fossil species from early Eocene South America. Am J Bot 107: 1749–1762 33247843 10.1002/ajb2.1565

[koac305-B16] Dobin A , Davis CA, Schlesinger F, Drenkow J, Zaleski C, Jha S, Batut P, Chaisson M, Gingeras TR (2013) STAR: ultrafast universal RNA-seq aligner. Bioinformatics 29: 15–21 23104886 10.1093/bioinformatics/bts635 PMC3530905

[koac305-B17] Dudchenko O , Shamim MS, Batra SS, Durand NC, Musial NT, Mostofa R, Pham M, Glenn St Hilaire B, Yao W, Stamenova E, et al (2018) The Juicebox Assembly Tools module facilitates novo; assembly of mammalian genomes with chromosome-length scaffolds for under $1000. BioRxiv, 254797, 10.1101/254797

[koac305-B19] Gao H , Li J, Wang L, Zhang J, He C (2020) Transcriptomic variation of the flower–fruit transition in Physalis and Solanum. Planta 252: 28 32720160 10.1007/s00425-020-03434-x

[koac305-B20] Gao L , Gonda I, Sun H, Ma Q, Bao K, Tieman DM, Burzynski-Chang EA, Fish TL, Stromberg KA, Sacks GL, et al (2019) The tomato pan-genome uncovers new genes and a rare allele regulating fruit flavor. Nat Genet 51: 1044–1051 31086351 10.1038/s41588-019-0410-2

[koac305-B21] Garrison EP , Marth GT (2012) Haplotype-based variant detection from short-read sequencing. ArXiv: Genomics. 10.48550/arXiv.1207.3907

[koac305-B22] Gebhardt C (2016) The historical role of species from the Solanaceae plant family in genetic research. Theor Appl Genet 129: 2281–2294 27744490 10.1007/s00122-016-2804-1 PMC5121179

[koac305-B23] Gilchrist CLM , Chooi YH (2021) clinker clustermap.js: automatic generation of gene cluster comparison figures. Bioinformatics 37: 2473–2475 10.1093/bioinformatics/btab007 33459763

[koac305-B24] Grandillo S , Tanksley SD (1996) QTL analysis of horticultural traits differentiating the cultivated tomato from the closely related species Lycopersicon pimpinellifolium. Theor Appl Genet 92: 935–951 24166620 10.1007/BF00224033

[koac305-B25] Guan D , McCarthy SA, Wood J, Howe K, Wang Y, Durbin R (2020) Identifying and removing haplotypic duplication in primary genome assemblies. Bioinformatics 36: 2896–2898 31971576 10.1093/bioinformatics/btaa025 PMC7203741

[koac305-B26] He C , Münster T, Saedler H (2004) On the origin of floral morphological novelties. FEBS Lett 567: 147–151 15165908 10.1016/j.febslet.2004.02.090

[koac305-B27] He C , Saedler H (2005) Heterotopic expression of MPF2 is the key to the evolution of the Chinese lantern of Physalis, a morphological novelty in Solanaceae. Proc Natl Acad Sci USA 102: 5779–5784 15824316 10.1073/pnas.0501877102 PMC556287

[koac305-B28] He C , Saedler H (2007) Hormonal control of the inflated calyx syndrome, a morphological novelty, in Physalis. Plant J 49: 935–946 17316177 10.1111/j.1365-313X.2006.03008.x

[koac305-B29] Hendelman A , Zebell S, Rodriguez-Leal D, Dukler N, Robitaille G, Wu X, Kostyun J, Tal L, Wang P, Bartlett ME, et al (2021) Conserved pleiotropy of an ancient plant homeobox gene uncovered by cis-regulatory dissection. Cell 184: 1724–1739.e16 33667348 10.1016/j.cell.2021.02.001

[koac305-B30] Hosmani PS , Flores-Gonzalez M, van de Geest H, Maumus F, Bakker LV, Schijlen E, van Haarst J, Cordewener J, Sanchez-Perez G, Peters S, et al (2019) An improved de novo assembly and annotation of the tomato reference genome using single-molecule sequencing, Hi-C proximity ligation and optical maps. BioRxiv, 767764. 10.1101/767764

[koac305-B31] Hu JY , Saedler H (2007) Evolution of the inflated calyx syndrome in Solanaceae. Mol Biol Evol 24: 2443–2453 17827172 10.1093/molbev/msm177

[koac305-B32] Huang M , He JX, Hu HX, Zhang K, Wang XN, Zhao BB, Lou HX, Ren DM, Shen T (2020) Withanolides from the genus Physalis: a review on their phytochemical and pharmacological aspects. J Pharm Pharmacol 72: 649–669 31826333 10.1111/jphp.13209

[koac305-B33] Itkin M , Seybold H, Breitel D, Rogachev I, Meir S, Aharoni A (2009) TOMATO AGAMOUS-LIKE 1 is a component of the fruit ripening regulatory network. Plant J 60: 1081–1095 19891701 10.1111/j.1365-313X.2009.04064.x

[koac305-B34] Jain C , Rhie A, Hansen N, Koren S, Phillippy AM (2020a) A long read mapping method for highly repetitive reference sequences. BioRxiv, 2020.11.01.363887, 10.1101/2020.11.01.363887 PMC10510034 35365778

[koac305-B35] Jain C , Rhie A, Zhang H, Chu C, Walenz BP, Koren S, Phillippy AM (2020b) Weighted minimizer sampling improves long read mapping. Bioinformatics 36(Suppl 1): i111–i118 32657365 10.1093/bioinformatics/btaa435 PMC7355284

[koac305-B36] Katoh K , Standley DM (2013) MAFFT multiple sequence alignment software version 7: improvements in performance and usability. Mol Biol Evol 30: 772–780 23329690 10.1093/molbev/mst010 PMC3603318

[koac305-B37] Kim S , Park M, Yeom SI, Kim YM, Lee JM, Lee HA, Seo E, Choi J, Cheong K, Kim KT, et al (2014) Genome sequence of the hot pepper provides insights into the evolution of pungency in Capsicum species. Nat Genet 46: 270–278 24441736 10.1038/ng.2877

[koac305-B38] Kirsche M , Prabhu G, Sherman R, Ni B, Aganezov S, Schatz MC (2021) Jasmine: population-scale structural variant comparison and analysis. BioRxiv, 2021.05.27.445886. 10.1038/s41592-022-01753-3 PMC10006329 36658279

[koac305-B39] Kolmogorov M , Yuan J, Lin Y, Pevzner PA (2019) Assembly of long, error-prone reads using repeat graphs. Nat Biotechnol 37: 540–546 30936562 10.1038/s41587-019-0072-8

[koac305-B40] Kurtz S , Phillippy A, Delcher AL, Smoot M, Shumway M, Antonescu C, Salzberg SL (2004) Versatile and open software for comparing large genomes. Genome Biol 5: R12 14759262 10.1186/gb-2004-5-2-r12 PMC395750

[koac305-B41] Kwon CT , Tang L, Wang X, Gentile I, Hendelman A, Robitaille G, Van Eck J, Xu C, Lippman ZB (2022) Dynamic evolution of small signalling peptide compensation in plant stem cell control. Nat Plants 8: 346–355 35347264 10.1038/s41477-022-01118-w

[koac305-B42] Lemmon ZH , Reem NT, Dalrymple J, Soyk S, Swartwood KE, Rodriguez-Leal D, van Eck J, Lippman ZB (2018) Rapid improvement of domestication traits in an orphan crop by genome editing. Nat Plants 4: 766–770 30287957 10.1038/s41477-018-0259-x

[koac305-B43] Li H (2013) Aligning sequence reads, clone sequences and assembly contigs with BWA-MEM. arXiv: 1303.3997v2 [q-bio.GN]

[koac305-B44] Li H (2018) Minimap2: pairwise alignment for nucleotide sequences. Bioinformatics 34: 3094–3100 29750242 10.1093/bioinformatics/bty191 PMC6137996

[koac305-B45] Li H , Handsaker B, Wysoker A, Fennell T, Ruan J, Homer N, Marth G, Abecasis G, Durbin R, Subgroup, 1000 Genome Project Data Processing (2009) The sequence alignment/map format and SAMtools. Bioinformatics 25: 2078–2079 19505943 10.1093/bioinformatics/btp352 PMC2723002

[koac305-B46] Li J , Song C, He C (2019) Chinese lantern in Physalis is an advantageous morphological novelty and improves plant fitness. Sci Rep 9: 596 30679462 10.1038/s41598-018-36436-7 PMC6345875

[koac305-B47] Liu Y , Tikunov Y, Schouten RE, Marcelis LFM, Visser RGF, Bovy A (2018) Anthocyanin biosynthesis and degradation mechanisms in Solanaceous vegetables: a review. Front Chem 6: 52 29594099 10.3389/fchem.2018.00052 PMC5855062

[koac305-B48] Love MI , Huber W, Anders S (2014) Moderated estimation of fold change and dispersion for RNA-seq data with DESeq2. Genome Biol 15: 550 25516281 10.1186/s13059-014-0550-8 PMC4302049

[koac305-B49] Lu J , Luo M, Wang L, Li K, Yu Y, Yang W, Gong P, Gao H, Li Q, Zhao J, et al (2021) The *Physalis floridana* genome provides insights into the biochemical and morphological evolution of Physalis fruits. Hortic Res 8: 244 34795210 10.1038/s41438-021-00705-w PMC8602270

[koac305-B50] Mapleson D , Venturini L, Kaithakottil G, Swarbreck D (2018) Efficient and accurate detection of splice junctions from RNA-seq with Portcullis. GigaScience 7: giy131 30418570 10.1093/gigascience/giy131 PMC6302956

[koac305-B51] Marini F , Binder H (2019) pcaExplorer: an R/Bioconductor package for interacting with RNA-seq principal components. BMC Bioinform 20: 331 10.1186/s12859-019-2879-1 PMC6567655 31195976

[koac305-B52] Martínez M (1993) The correct application of *Physalis pruinosa* L. (Solanaceae). Taxon 42: 103–104

[koac305-B53] Meir Z , Aviezer I, Chongloi GL, Ben-Kiki O, Bronstein R, Mukamel Z, Keren-Shaul H, Jaitin D, Tal L, Shalev-Schlosser G, et al (2021) Dissection of floral transition by single-meristem transcriptomes at high temporal resolution. Nat Plants 7: 800–813 34135484 10.1038/s41477-021-00936-8

[koac305-B54] Minh BQ , Schmidt HA, Chernomor O, Schrempf D, Woodhams MD, von Haeseler A, Lanfear R (2020) IQ-TREE 2: new models and efficient methods for phylogenetic inference in the genomic eEra. Mol Biol Evol 37: 1530–1534 32011700 10.1093/molbev/msaa015 PMC7182206

[koac305-B55] Morel P , Heijmans K, Rozier F, Zethof J, Chamot S, Bento SR, Vialette-Guiraud A, Chambrier P, Trehin C, Vandenbussche M (2017) Divergence of the floral a-function between an asterid and a rosid species. Plant Cell 29: 1605–1621 28646074 10.1105/tpc.17.00098 PMC5559753

[koac305-B56] Morgulis A , Gertz EM, Schäffer AA, Agarwala R (2006) WindowMasker: window-based masker for sequenced genomes. Bioinformatics 22: 134–141 16287941 10.1093/bioinformatics/bti774

[koac305-B57] Muller GB , Wagner GP (1991) Novelty in evolution: restructuring the concept. Ann Rev Ecol Syst 22: 229–256

[koac305-B58] Nurk S , Walenz BP, Rhie A, Vollger MR, Logsdon GA, Grothe R, Miga KH, Eichler EE, Phillippy AM, Koren S (2020) HiCanu: accurate assembly of segmental duplications, satellites, and allelic variants from high-fidelity long reads. Genome Res 30: 1291–1305 32801147 10.1101/gr.263566.120 PMC7545148

[koac305-B59] Ou S , Su W, Liao Y, Chougule K, Agda JRA, Hellinga AJ, Lugo CSB, Elliott TA, Ware D, Peterson T, et al (2019) Benchmarking transposable element annotation methods for creation of a streamlined, comprehensive pipeline. Genome Biol 20: 275 31843001 10.1186/s13059-019-1905-y PMC6913007

[koac305-B60] Padmaja H , Sruthi SR, Vangalapati M (2014) Review on Hibiscus sabdariffa - A valuable herb. Int J Pharm Life Sci 5: 3747–3752

[koac305-B61] Pan IL , McQuinn R, Giovannoni JJ, Irish VF (2010) Functional diversification of AGAMOUS lineage genes in regulating tomato flower and fruit development. J Exp Bot 61: 1795–1806 20335407 10.1093/jxb/erq046 PMC2852668

[koac305-B62] Park SJ , Eshed Y, Lippman ZB (2014) Meristem maturation and inflorescence architecture—lessons from the Solanaceae. Curr Opin Plant Biol 17: 70–77 24507497 10.1016/j.pbi.2013.11.006

[koac305-B63] Paton A (1990) A global taxonomic investigation of Scutellaria (Labiatae). Kew Bull 45 399–450

[koac305-B64] Patro R , Duggal G, Love MI, Irizarry RA, Kingsford C (2017) Salmon provides fast and bias-aware quantification of transcript expression. Nat Methods 14: 417–419 28263959 10.1038/nmeth.4197 PMC5600148

[koac305-B65] Pertea G , Pertea M (2020) GFF utilities: GffRead and GffCompare. F1000Res 9: 304 10.12688/f1000research.23297.1 PMC7222033 32489650

[koac305-B66] Pertea M , Pertea GM, Antonescu CM, Chang TC, Mendell JT, Salzberg SL (2015) StringTie enables improved reconstruction of a transcriptome from RNA-seq reads. Nat Biotechnol 33: 290–295 25690850 10.1038/nbt.3122 PMC4643835

[koac305-B67] Picard Toolkit (2019) GitHub Repository. Broad Institute, Cambridge, MA

[koac305-B68] Pnueli L , Hareven D, Rounsley SD, Yanofsky MF, Lifschitz E (1994) Isolation of the tomato AGAMOUS gene TAG1 and analysis of its homeotic role in transgenic plants. Plant Cell 6: 163–173 7908549 10.1105/tpc.6.2.163 PMC160424

[koac305-B69] Pretz C , Deanna R (2020) Typifications and nomenclatural notes in Physalis (Solanaceae) from the United States. Taxon 69: 170–192

[koac305-B70] R Core Team (2020) R: A Language and Environment for Statistical Computing. R Core Team, Vienna, Austria

[koac305-B71] Rhie A , McCarthy SA, Fedrigo O, Damas J, Formenti G, Koren S, Uliano-Silva M, Chow W, Fungtammasan A, Kim J, et al (2021) Towards complete and error-free genome assemblies of all vertebrate species. Nature 592: 737–746 33911273 10.1038/s41586-021-03451-0 PMC8081667

[koac305-B72] Rhie A , Walenz BP, Koren S, Phillippy AM (2020) Merqury: reference-free quality, completeness, and phasing assessment for genome assemblies. Genome Biol 21: 245 32928274 10.1186/s13059-020-02134-9 PMC7488777

[koac305-B73] Rydberg PA (1896) The North American Species of Physalis and Related Genera. Torrey Botanical Club, New York, NY

[koac305-B74] Särkinen T , Bohs L, Olmstead RG, Knapp S (2013) A phylogenetic framework for evolutionary study of the nightshades (Solanaceae): a dated 1000-tip tree. BMC Evol Biol 13: 214 24283922 10.1186/1471-2148-13-214 PMC3850475

[koac305-B75] Sato S , Tabata S, Hirakawa H, Asamizu E, Shirasawa K, Isobe S, Kaneko T, Nakamura Y, Shibata D, Aoki K, et al (2012) The tomato genome sequence provides insights into fleshy fruit evolution. Nature 485: 635–641 22660326 10.1038/nature11119 PMC3378239

[koac305-B76] Senthil-Kumar M , Mysore KS (2011) Caveat of RNAi in plants: the off-target effect. *In* Kodama H, Komamine A, eds, RNAi and Plant Gene Function Analysis: Methods and Protocols. Humana Press, Totowa, NJ, pp 13–25 10.1007/978-1-61779-123-9_2 21533683

[koac305-B77] Shenstone E , Lippman Z, van Eck J (2020) A review of nutritional properties and health benefits of physalis species. Plant Foods Hum Nutr 75: 316–325 32385801 10.1007/s11130-020-00821-3

[koac305-B78] Shubin N , Tabin C, Carroll S (2009) Deep homology and the origins of evolutionary novelty. Nature 457: 818–823 19212399 10.1038/nature07891

[koac305-B79] Shumate A , Salzberg SL (2021) Liftoff: accurate mapping of gene annotations. Bioinformatics 37: 1639–1643 10.1093/bioinformatics/btaa1016 PMC8289374 33320174

[koac305-B80] Simão FA , Waterhouse RM, Ioannidis P, Kriventseva EV, Zdobnov EM (2015) BUSCO: assessing genome assembly and annotation completeness with single-copy orthologs. Bioinformatics 31: 3210–3212 26059717 10.1093/bioinformatics/btv351

[koac305-B81] Soneson C , Love MI, Robinson MD (2016) Differential analyses for RNA-seq: transcript-level estimates improve gene-level inferences. F1000Res 4: 1521 10.12688/f1000research.7563.1 PMC4712774 26925227

[koac305-B82] Soyk S , Lemmon ZH, Oved M, Fisher J, Liberatore KL, Park SJ, Goren A, Jiang K, Ramos A, van der Knaap E, et al (2017) Bypassing negative epistasis on yield in tomato imposed by a domestication gene. Cell 169: 1142–1155.e12 28528644 10.1016/j.cell.2017.04.032

[koac305-B83] Spelt C , Quattrocchio F, Mol J, Koes R (2002) ANTHOCYANIN1 of petunia controls pigment synthesis, vacuolar pH, and seed coat development by genetically distinct mechanisms. Plant Cell 14: 2121–2135 12215510 10.1105/tpc.003772 PMC150760

[koac305-B84] Spelt C , Quattrocchio F, Mol JNM, Koes R (2000) anthocyanin1 of Petunia encodes a basic helix-loop-helix protein that directly activates transcription of structural anthocyanin genes. Plant Cell 12: 1619–1631 11006336 10.1105/tpc.12.9.1619 PMC149074

[koac305-B85] Sugiyama Y , Watase Y, Nagase M, Makita N, Yagura S, Hirai A, Sugiura M (2005) The complete nucleotide sequence and multipartite organization of the tobacco mitochondrial genome: comparative analysis of mitochondrial genomes in higher plants. Mol Genetics Genomics 272: 603–615 10.1007/s00438-004-1075-8 15583938

[koac305-B86] Swartwood K , van Eck J (2019) Development of plant regeneration and *Agrobacterium tumefaciens*-mediated transformation methodology for *Physalis pruinosa*. Plant Cell Tissue Organ Culture 137: 465–472

[koac305-B87] Takagi H , Abe A, Yoshida K, Kosugi S, Natsume S, Mitsuoka C, Uemura A, Utsushi H, Tamiru M, Takuno S, et al (2013) QTL-seq: rapid mapping of quantitative trait loci in rice by whole genome resequencing of DNA from two bulked populations. Plant J 74: 174–183 23289725 10.1111/tpj.12105

[koac305-B88] Tamura K , Stecher G, Kumar S (2021) MEGA11: molecular evolutionary genetics analysis version 11. Mol Biol Evol 38: 3022–3027 33892491 10.1093/molbev/msab120 PMC8233496

[koac305-B89] Theißen G , Saedler H (2001) Floral quartets. Nature 409: 469–471 10.1038/35054172 11206529

[koac305-B90] Venturini L , Caim S, Kaithakottil GG, Mapleson DL, Swarbreck D (2018) Leveraging multiple transcriptome assembly methods for improved gene structure annotation. GigaScience 7: giy093 30052957 10.1093/gigascience/giy093 PMC6105091

[koac305-B91] Waterfall UT (1967) Physalis in Mexico, Central America and the West Indies. Rhodora 69: 82–120

[koac305-B92] Waterfall UT , Umaldy T (1958) A taxonomic study of the genus Physalis in North America north of Mexico. Rhodora 60: 152–173

[koac305-B93] Wei Q , Wang J, Wang W, Hu T, Hu H, Bao C (2020) A high-quality chromosome-level genome assembly reveals genetics for important traits in eggplant. Hortic Res 7: 153 33024567 10.1038/s41438-020-00391-0 PMC7506008

[koac305-B94] Weigel D , Meyerowitz EM (1994) The ABCs of floral homeotic genes. Cell 78: 203–209 7913881 10.1016/0092-8674(94)90291-7

[koac305-B95] Whitson M (2012) Calliphysalis (solanaceae): a new genus from the southeastern USA. Rhodora 114: 133–147

[koac305-B96] Wilf P , Carvalho MR, Gandolfo MA, Cúneo NR (2017) Eocene lantern fruits from Gondwanan Patagonia and the early origins of Solanaceae. Science 355: 71–75 28059765 10.1126/science.aag2737

[koac305-B97] Xu P , Zhang Y, Kang L, Roossinck MJ, Mysore KS (2006) Computational estimation and experimental verification of off-target silencing during posttranscriptional gene silencing in plants. Plant Physiol 142: 429–440 16920874 10.1104/pp.106.083295 PMC1586062

[koac305-B98] Xu X , Pan S, Cheng S, Zhang B, Mu D, Ni P, Zhang G, Yang S, Li R, Wang J, et al (2011) Genome sequence and analysis of the tuber crop potato. Nature 475: 189–195 21743474 10.1038/nature10158

[koac305-B99] Yanofsky MF , Ma H, Bowman JL, Drews GN, Feldmann KA, Meyerowitz EM (1990) The protein encoded by the Arabidopsis homeotic gene agamous resembles transcription factors. Nature 346: 35–39 1973265 10.1038/346035a0

[koac305-B100] Yuste-Lisbona FJ , Quinet M, Fernández-Lozano A, Pineda B, Moreno V, Angosto T, Lozano R (2016) Characterization of vegetative inflorescence (mc-vin) mutant provides new insight into the role of MACROCALYX in regulating inflorescence development of tomato. Sci Rep 6: 18796 26727224 10.1038/srep18796 PMC4698712

[koac305-B101] Zamora-Tavares M del P , Martínez M, Magallón S, Guzmán-Dávalos L, Vargas-Ponce O (2016) Physalis and physaloids: a recent and complex evolutionary history. Mol Phylogenet Evol 100: 41–50 27063196 10.1016/j.ympev.2016.03.032

[koac305-B102] Zhang JS , Li Z, Zhao J, Zhang S, Quan H, Zhao M, He C (2014) Deciphering the *Physalis floridana* double-layered-lantern1 mutant provides insights into functional divergence of the GLOBOSA duplicates within the Solanaceae. Plant Physiol 164: 748–764 24390390 10.1104/pp.113.233072 PMC3912103

[koac305-B103] Zhang WN , Tong WY (2016) Chemical constituents and biological activities of plants from the genus physalis. Chem Biodivers 13: 48–65 26765352 10.1002/cbdv.201400435

[koac305-B104] Zhao J , Tian Y, Zhang JS, Zhao M, Gong P, Riss S, Saedler R, He C (2013) The euAP1 protein MPF3 represses MPF2 to specify floral calyx identity and displays crucial roles in Chinese lantern development in Physalis. Plant Cell 25: 2002–2021 23792370 10.1105/tpc.113.111757 PMC3723609

