# Supplementary material for: Establishing Physalis as a Solanaceae model system enables genetic reevaluation of the inflated calyx syndrome
Source: Plant Cell. 2022 Oct 21;35(1):351–68. doi: 10.1093/plcell/koac305 (PMC9806562; doi:10.1093/plcell/koac305)
Supplement: koac305_Supplementary_Data [file koac305_supplementary_data.zip › SupplementaryFile/tpc.22.00728Supplemental Figures and Tables.pdf]

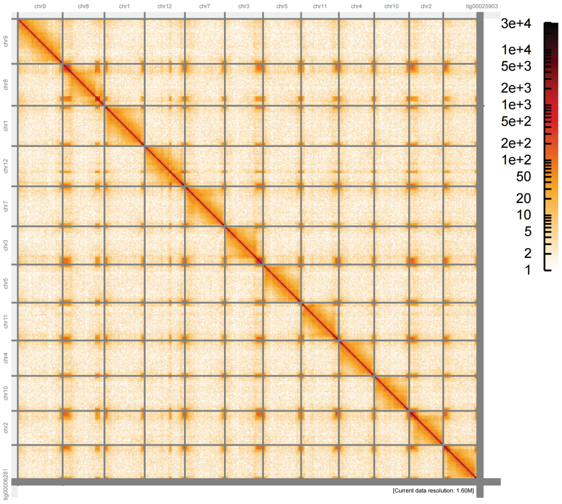

P. grisea

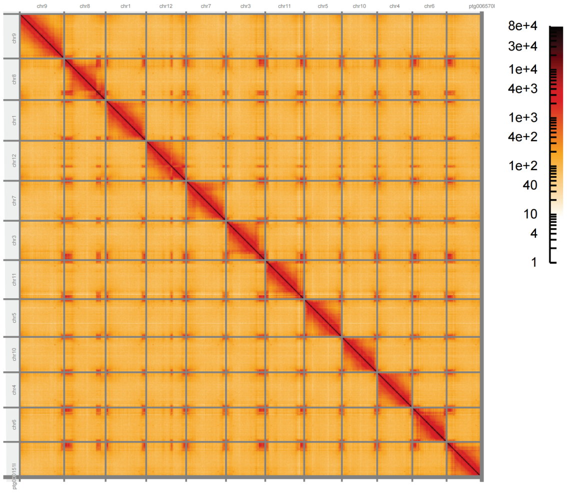

P. pruinosa

Supplemental Figure S1 Hi-C heatmaps confirm reference assembly structural accuracy. (Supports **Figure 1**)
Hi-C heatmaps for the *P. grisea* and *P. pruinosa* reference assemblies. The 12 chromosomes are sorted from largest (top left) to smallest (bottom right).

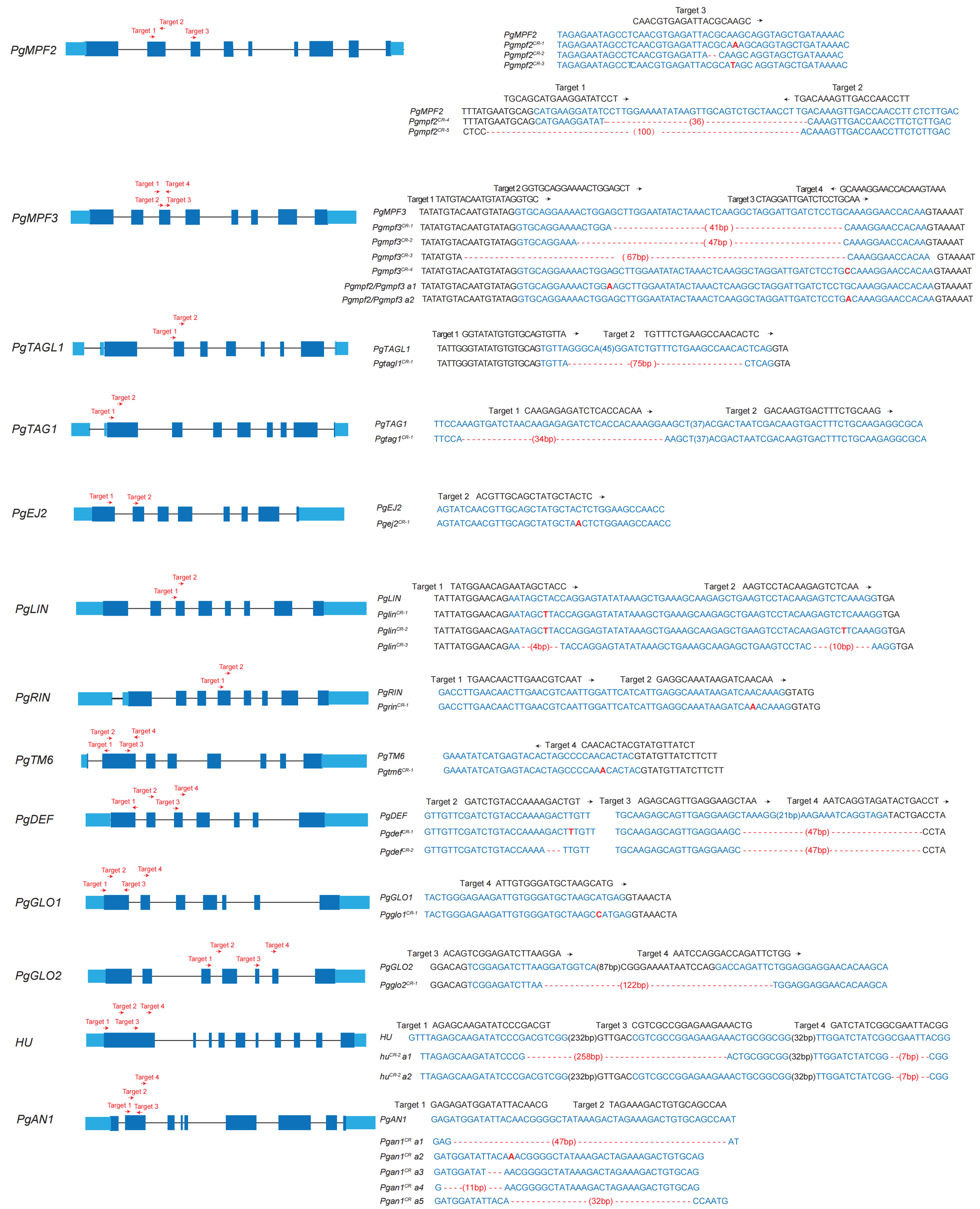
PgMPF2
PgMPF3
PgTAGL1
PgTAG1
PgEJ2
PgLIN
PgRIN
PgTM6
PgDEF
PgGLO1
PgGLO2
HU
PgAN1

Supplemental Figure S2. Illustrations of CRISPR-engineered mutations in this study. (Supports **Figure 2, 3, 4, 5**)
In all gene models, deep blue boxes, black lines, and light blue boxes represent exonic, intronic, and untranslated regions, respectively. Red dashed lines indicate indels/deletions; Red bold nucleotides represent inserts.

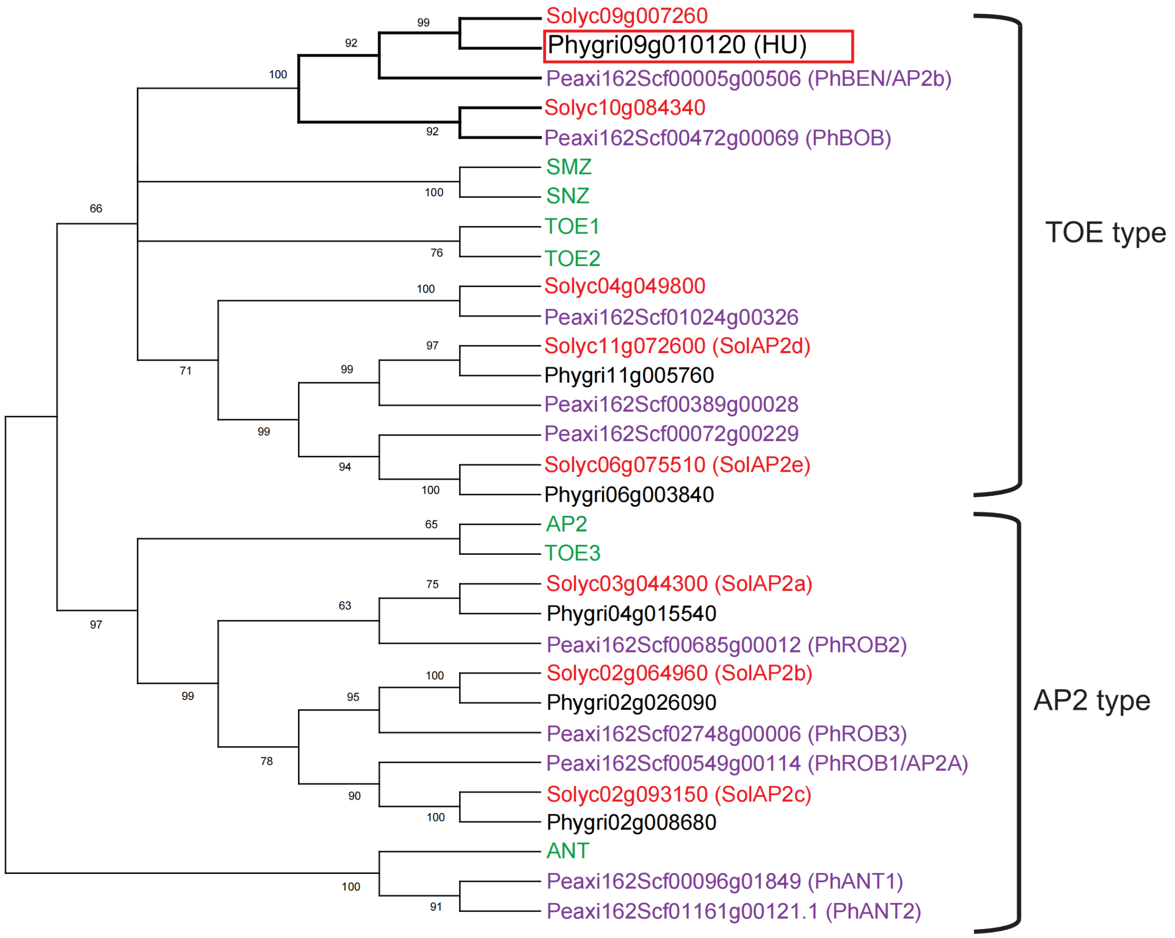

Supplemental Figure S3. Maximum Likelihood consensus tree of the euAP2 proteins from *A. thaliana* (gene names in green), *P. axillaris* (Peaxi IDs in purple), *S. lycopersicum* (Solyc IDs in red), and *P. grisea* (Phygri IDs in black). (Supports **Figure 5**). Bootstrap values (%) based on 500 replicates are indicated near the branching points; branches below 50% have been collapsed. Protein sequences used to build the tree can be found in **Supplemental Data Set S7**.

Supplemental Table S1. Genome assembly statistics.

sample	assembly	# sequences	assembly size (bp)	maximum contig size (bp)
P. pruinosa	contigs	254	1,384,030,950	119,196,254
P. pruinosa	scaffolds	133	1,384,506,225	132,724,694
P. grisea	contigs	1,007	1,367,029,362	72,969,772
P. grisea	scaffolds	460	1,373,559,025	132,441,007
P. floridana	contigs	948	1,389,237,711	21,252,035
P. floridana	scaffolds	327	1,389,299,811	149,502,688

sample	assembly	auN	N50 (bp)	N90 (bp)	L50	L90	Merqury QV
P. pruinosa	contigs	76,798,857	82,161,017	16,197,355	7	21	NA
P. pruinosa	scaffolds	115,087,539	117,388,568	102,489,187	6	11	57.8095
P. grisea	contigs	35,675,292	31,630,509	6,451,401	13	49	NA
P. grisea	scaffolds	112,605,104	112,874,881	100,751,416	6	11	55.1067
P. floridana	contigs	5,562,125	4,584,214	1,040,892	95	325	NA
P. floridana	scaffolds	116,200,624	113,373,559	96,490,955	6	11	34.1672

sample	assembly	Merqury Completeness (%)	Complete BUSCO genome (%)	Complete BUSCO proteins (%)
P. pruinosa	contigs	NA	NA	NA
P. pruinosa	scaffolds	98.2754	97.9	96.9
P. grisea	contigs	NA	NA	NA
P. grisea	scaffolds	98.9148	97.9	97.2
P. floridana	contigs	NA	NA	NA
P. floridana	scaffolds	94.8237	97.9	90.1

sample	assembly	# C.annuum transcripts aligned	Median C.annuum transcript alignment identity (%)
P. pruinosa	contigs	NA	NA
P. pruinosa	scaffolds	25652	89.5126
P. grisea	contigs	NA	NA
P. grisea	scaffolds	25619	89.5199
P. floridana	contigs	NA	NA
P. floridana	scaffolds	26096	89.5833

Supplemental Table S2. Annotation stats of *P. grisea* and *P. pruinosa* genomes.

	P. grisea	***P. pruinosa***
Number of genes	33833	34187
Number of mrnas	33849	34188
Number of mrnas with utr both sides	26250	26372
Number of mrnas with at least one utr	30519	30730
Number of cdss	33459	33786
Number of exons	158014	158046
Number of five_prime_utrs	27706	27860
Number of three_prime_utrs	29063	29242
Number of exon in cds	145780	145852
Number of exon in five_prime_utr	34944	35055
Number of exon in three_prime_utr	33307	33455
Number of intron in cds	112321	112066
Number of intron in exon	124181	123859
Number of intron in five_prime_utr	7238	7195
Number of intron in three_prime_utr	4244	4213
Number gene overlapping	1956	1961
Number of single exon gene	7473	7843
Number of single exon mrna	7473	7843
mean mrnas per gene	1	1
mean cdss per mrna	1	1
mean exons per mrna	4.7	4.6
mean five_prime_utrs per mrna	0.8	0.8
mean three_prime_utrs per mrna	0.9	0.9
mean exons per cds	4.4	4.3
mean exons per five_prime_utr	1.3	1.3
mean exons per three_prime_utr	1.1	1.1
mean introns in cdss per mrna	3.3	3.3
mean introns in exons per mrna	3.7	3.6
mean introns in five_prime_utrs per mrna	0.2	0.2
mean introns in three_prime_utrs per mrna	0.1	0.1
Total gene length	161975583	158646609
Total mrna length	162000966	158647900
Total cds length	33946614	33901244
Total exon length	47579508	47550517
Total five_prime_utr length	4887501	4886475
Total three_prime_utr length	8677404	8690880
Total intron length per cds	97759857	94545905
Total intron length per exon	114520256	111219951
Total intron length per five_prime_utr	10121209	9341900
Total intron length per three_prime_utr	8061745	6840589

mean gene length	4787	4640
mean mrna length	4785	4640
mean cds length	1014	1003
mean exon length	301	300
mean five_prime_utr length	176	175
mean three_prime_utr length	298	297
mean cds piece length	232	232
mean five_prime_utr piece length	139	139
mean three_prime_utr piece length	260	259
mean intron in cds length	870	843
mean intron in exon length	922	897
mean intron in five_prime_utr length	1398	1298
mean intron in three_prime_utr length	1899	1623
Longest gene	1021807	1021806
Longest mrna	1021807	1021806
Longest cds	16299	16299
Longest exon	13821	11707
Longest five_prime_utr	7342	7342
Longest three_prime_utr	11757	6272
Longest cds piece	7935	7935
Longest five_prime_utr piece	6904	6904
Longest three_prime_utr piece	11506	6272
Longest intron into cds part	656789	150320
Longest intron into exon part	1021346	1021345
Longest intron into five_prime_utr part	320971	109086
Longest intron into three_prime_utr part	1021346	1021345
Shortest gene	37	37
Shortest mrna	37	37
Shortest cds	12	12
Shortest exon	1	1
Shortest five_prime_utr	1	1
Shortest three_prime_utr	1	1
Shortest cds piece	1	1
Shortest five_prime_utr piece	1	1
Shortest three_prime_utr piece	1	1
Shortest intron into cds part	2	2
Shortest intron into exon part	2	2
Shortest intron into five_prime_utr part	2	2
Shortest intron into three_prime_utr part	2	2
Note: stats based on annotation *P. gri* v1.3.2 and *P. pru* v2.0.4		

Supplemental Table S3. Result summary of SNP calls of *P. pruinosa* Illumina reads against *P. grisea* as reference.

SNP Type	Count	Percent
3 prime UTR variant	399	0.44%
5 prime UTR premature start codon gain variant	23	0.03%
5 prime UTR variant	237	0.26%
downstream gene variant	15,043	16.54%
initiator codon variant	1	0.00%
intergenic region	53,865	59.21%
intron variant	4,443	4.88%
missense variant	798	0.88%
non coding transcript exon variant	202	0.22%
splice acceptor variant	2	0.00%
splice donor variant	4	0.00%
splice region variant	68	0.08%
stop gained	9	0.01%
stop lost	30	0.03%
stop retained variant	1	0.00%
synonymous variant	472	0.52%
upstream gene variant	15,381	16.91%
**SNP region**	**Count**	**Percent**
downstream	15,043	16.55%
exon	1,504	1.65%
intergenic	53,865	59.25%
intron	4,387	4.83%
splice site acceptor	2	0.00%
splice site donor	4	0.00%
splice site region	65	0.07%
upstream	15,381	16.92%
utr 3 prime	399	0.44%
utr 5 prime	260	0.29%
**Exonic SNP effect Type**	**Count**	**Percent**
missense	829	63.23%
nonsense	9	0.69%
silent	473	36.08%

Supplemental Table S4. High impact SNP calls of *P. pruinosa* Illumina reads against *P. grisea* as reference.

Phygri ID	SNP in *P. pru*	Variant effect type	Gene annotation
Phygri02g019330	T to C	stop lost	ATP synthase subunit beta
Phygri02g026730	C to T	stop gain	Photosystem II protein D1 (AHRD V3.3 *-* A0A1U8QBZ9_CAPAN)
Phygri02g028210	C to G	stop lost	Protein Ycf2 (AHRD V3.3 *-* A0A2G2VDC7_CAPBA)
Phygri02g028212	T to G	stop lost	Protein Ycf2 (AHRD V3.3 *-* A0A2G2XBS0_CAPBA)
Phygri02g028890	T to C	stop lost	Unknown protein
Phygri03g006170	C to G	stop gain	NADH-ubiquinone oxidoreductase chain 5
Phygri03g006220	T to C	stop lost	Ribosomal protein S4 (AHRD V3.3 *-* A0A0C5B2D1_HYONI)
Phygri03g008070	T to C	stop lost	Cytochrome c biogenesis FC (AHRD V3.3 *-* A0A0C5APX0_HYONI)
Phygri03g008080	T to C	stop lost	Cytochrome c biogenesis FC (AHRD V3.3 *-* A0A0C5B7H6_9SOLA)
Phygri03g008100	T to C	stop lost	Unknown protein
Phygri03g008140	T to C	stop lost	Unknown protein
Phygri03g008210	A to T	stop lost	"ATP synthase subunit c", "chloroplastic (AHRD V3.3 *** A0A191T6I2_9VIRI)"
Phygri03g010620	T to A	stop gain	phytochrome C
Phygri03g020470	A to C	stop lost	ATP synthase subunit alpha (AHRD V3.3 *-* Q34265_CALUS)
Phygri05g004630	G to A	splice variant	Disease resistance protein (CC-NBS-LRR class) family
Phygri05g009410	T to A	stop lost	DNA-directed RNA polymerase subunit beta (AHRD V3.3 *-* A0A2G2V8P8_CAPBA)
Phygri05g009780	T to C	stop lost	DNA-directed RNA polymerase subunit beta" (AHRD V3.3 *-* A0A2H4N1U1_ARTAN)
Phygri05g010950	A to C	stop gain	Unknown protein
Phygri05g011012	G to A	splice variant	NA
Phygri06g001370	A to C	stop lost	O-methyltransferase family protein
Phygri06g002280	T to C	stop lost	ATP synthase subunit beta
Phygri06g018840	C to T	stop gain	Unknown protein
Phygri06g021920	A to G	stop lost	ATP synthase subunit beta (AHRD V3.3 *-* B3TLW1_ELAGV)
Phygri06g022110	T to C	stop lost	ATPase subunit 4 (AHRD V3.3 *-* A0A0C5B288_HYONI)
Phygri06g022220	T to G	stop lost	Transport membrane protein (AHRD V3.3 *-* A0A0C5ARU2_HYONI)
Phygri06g022240	G to C	splice variant	NADH dehydrogenase subunit 9 (AHRD V3.3 *** A0A290WKX8_SOLLC)
Phygri06g022360	A to C	stop lost	"Cytochrome C oxidase subunit II-like", "transmembrane domain-containing protein (AHRD V3.3 *** ATMG01280.1)"
Phygri06g027720	A to G	splice variant	cullin 1
Phygri07g011190	C to T	splice variant	2-oxoglutarate (2OG) and Fe(II)-dependent oxygenase superfamily protein
Phygri07g013270	A to G	stop lost	Cytochrome c oxidase subunit 3 (AHRD V3.3 *** A0A0C5BKQ0_9SOLA)
Phygri07g017780	A to T	stop gain	Proteinase inhibitor I3
Phygri07g021172	A to T	stop gain	NA

Phygri08g029160	A to T	stop lost	"DNA-directed RNA polymerase", " RpoA/D/Rpb3-type", " DNA-directed RNA polymerase", " insert domain", " DNA-directed RNA polymerase", " RBP11-like dimerisation domain"
Phygri08g033960	G to C	stop lost	NADH-ubiquinone oxidoreductase chain 5 (AHRD V3.3 *-* A0A0C5ARN6_HYONI)
Phygri08g044700	A to G	stop lost	Photosystem II CP43 reaction center protein (AHRD V3.3 *-* A0A0K0M2C6_9ASTR)
Phygri09g011240	T to A	stop gain	Unknown protein
Phygri10g009050	T to C	stop lost	Ribosomal protein L5 (AHRD V3.3 *** A0A290WL59_SOLLC)
Phygri11g007150	T to G	stop lost	"NAD(P)H-quinone oxidoreductase subunit K", " chloroplastic (AHRD V3.3 *-* I6N960_DATST)"
Phygri11g008530	A to C	stop lost	DNA-directed RNA polymerase subunit beta" (AHRD V3.3 *-* A0A2Z5TZR0_9ASPA)
Phygri11g012920	T to G	splice variant	embryo defective 2410
Phygri12g003540	T to A	stop lost	Calcium-binding mitochondrial carrier protein SCaMC-1-like (AHRD V3.3 *-* A0A2K3PR43_TRIPR)
Phygri12g012930	A to G	stop lost	Maturase K (AHRD V3.3 *** A0A2G3DGH8_CAPCH)
Phygri12g019240	A to C	stop gain	Unknown protein

Supplemental Table S5. SVs intersecting CDS.

Gene ID	Annotation Note
Phygri01g016370	Glutathione S-transferase family protein
Phygri02g002310	Secretory carrier membrane protein (SCAMP) family protein
Phygri02g002320	glycine-rich protein
Phygri02g002330	"3(2)", "5 -bisphosphate nucleotidase HAL2", " Inositol monophosphatase", " Inositol monophosphatase", " metal-binding site", " Inositol monophosphatase", " conserved site"
Phygri03g013930	Pyridoxal phosphate phosphatase-related protein
Phygri04g003300	Unknown protein
Phygri04g003302	NA
Phygri04g003304	NA
Phygri04g003310	Unknown protein
Phygri04g019590	Ribulose bisphosphate carboxylase large chain
Phygri05g004540	Disease resistance protein (CC-NBS-LRR class) family
Phygri05g004672	NA
Phygri05g010920	Unknown protein
Phygri05g012730	GDSL esterase/lipase
Phygri06g010820	Unknown protein
Phygri06g010830	Unknown protein
Phygri06g010840	DUF247 domain-containing protein (AHRD V3.3 *** A0A1Q3BSN5_CEPFO)
Phygri06g012120	2-oxoglutarate (2OG) and Fe(II)-dependent oxygenase superfamily protein
Phygri06g018140	Integrase-type DNA-binding superfamily protein
Phygri06g018450	The fantastic four family
Phygri07g006870	F-box protein (AHRD V3.3 *** A0A2K3P6V3_TRIPR)
Phygri07g020970	2-oxoglutarate (2OG) and Fe(II)-dependent oxygenase superfamily protein (AHRD V3.3 *** A0A2U1NTU8_ARTAN)
Phygri07g021960	Cytochrome P450 superfamily protein
Phygri07g022090	"Glycoside hydrolase", " superfamily", " X8", " Glycoside hydrolase", " family 17", " Glycoside hydrolase", " catalytic domain"
Phygri08g016200	RING/U-box superfamily protein
Phygri08g017160	1-phosphatidylinositol phosphodiesterase (AHRD V3.3 *** E5GC05_CUCME)
Phygri10g011680	Disease resistance-responsive (dirigent-like protein) family protein
Phygri10g011690	Disease resistance-responsive (dirigent-like protein) family protein
Phygri10g011692	NA
Phygri10g011700	Unknown protein
Phygri10g011710	Unknown protein
Phygri10g011720	Disease resistance-responsive (dirigent-like protein) family protein
Phygri10g012040	polyubiquitin 10
Phygri10g015250	2-oxoglutarate-dependent dioxygenase DAO
Phygri10g017262	NA
Phygri10g017270	Unknown protein
Phygri10g017280	Unknown protein
Phygri11g000880	c("Flavonoid 3", "5-hydroxylase 1")
Phygri11g013040	Unknown protein
Phygri11g013050	Unknown protein

Phygri11g013052	NA
Phygri11g013060	"Transposon", " En/Spm-like", " Transposase-associated domain protein (AHRD V3.3 *-* A0A2U1N4P4_ARTAN)"
Phygri11g013070	"Transposase", " Ptta/En/Spm", " plant"
Phygri11g013080	"Transposase", " Ptta/En/Spm", " plant"
Phygri11g013090	Kinesin-like protein
Phygri11g013100	Unknown protein
Phygri11g013120	Unknown protein
Phygri12g013870	Protein phosphatase inhibitor 2 (IPP-2)
Phygri12g016180	Retrovirus-related Pol polyprotein from transposon TNT 1-94
Phygri12g016440	TCP family transcription factor
Phygri00g000510	Non-symbiotic hemoglobin 1

Supplemental Table S6: SNPs with predicted high impact on transcripts of *huskless.*

Gene ID	Impact	SNP type	Variant effect	Annotation notes
Phygri03g003290	High	G to A	stop gained	F-box domain
Phygri06g022190	High	A to G	stop lost	NADH dehydrogenase subunit 7
Phygri06g025220	High	G to A	stop gained	TRIO/F-actin-binding protein
Phygri08g025050	High	C to T	splice acceptor variant&intron variant	RNA-binding (RRM/RBD/RNP motifs) family protein
Phygri09g010120	High	G to A	splice acceptor variant&intron variant	AP2/ERF domain
Phygri11g003880	High	G to A	splice donor variant&intron variant	transcription factor-related
Phygri12g018010	High	G to A	stop gained	Wall-associated receptor kinase-like 8
Phygri12g020720	High	G to A	stop gained	cultured cell-like protein

Supplemental Table S7: Co-segregation test of the G/A SNP in Phygri09g010120 and the huskless phenotype.

GeneID	Genotype	Phynotype	Number of plants
Phygri09g010120	G/G	WT	35
	G/A	WT	50
	A/A	*huskless*	36

Supplemental Table S8. Genes related to this study.

P. gri **gene ID**	**gene name**	***S. lyc*** **ortholog gene ID**	***S. lyc*** **gene name**
Phygri12g018350	*PgMPF3*	Solyc05g056620	*MACROCALYX (MC)*
Phygri11g023460	*PgMPF2*	Solyc04g076280	*SlMBP24/MPF2-like*
Phygri04g010290	*PgAN1*	Solyc09g065100	*bHLH transcription factor 150*
Phygri02g019350	*PgTAG1*	Solyc02g071730	*TOMATO AGAMOUS 1 (TAG1)*
Phygri07g005850	*PgTAGL1*	Solyc07g055920	*TOMATO AGAMOUS-LIKE 1 (TAGL1)*
Phygri07g015380	*PgLIN*	Solyc04g005320	*LONG INFLORESCENCE (LIN)*
Phygri12g018340	*PgRIN*	Solyc05g012020	*RIPENING INHIBITOR (RIN)*
Phygri03g020760	*PgEJ2*	Solyc03g114840	*ENHANCER of JOINTLESS2 (EJ2)*
Phygri02g012900	*PgTM6*	Solyc02g084630	*TM6/TDR6*
Phygri01g009190	*PgGLO1*	Solyc08g067230	*GLOBOSA1 (SlGLO1)*
Phygri06g017940	*PgGLO2*	Solyc06g059970	*GLOBOSA2 (SlGLO2)/TPI*
Phygri11g018450	*PgDEF*	Solyc04g081000	*Deficiens (SlDEF)*
Phygri09g010120	*HUSKLESS*	Solyc09g007260	

Supplemental Table S9. CRISPR guides used in this study.

P. gri gene ID	gene name	CRISPR guide name	CRISPR guide sequence 5'-3'
Phygri12g018350	*PgMPF3*	PgMPF3-G1	TATGTACAATGTATAGGTGC
		PgMPF3-G2	GGTGCAGGAAAACTGGAGCT
		PgMPF3-G3	CTAGGATTGATCTCCTGCAA
		PgMPF3-G4	TTTACTTGTGGTTCCTTTGC
Phygri11g023460	*PgMPF2*	PgMPF2-G1	ATATATTATTGAGGATGGGA
		PgMPF2-G2	ATCTACTAAATTTACTAGCT
		PgMPF2-G3	TGCAGCATGAAGGATATCCT
		PgMPF2-G4	AAGGTTGGTCAACTTTGTCA
		PgMPF2-G5	GATTATATAAGCAACACAAA
		PgMPF2-G6	CAACGTGAGATTACGCAAGC
Phygri02g019350	*PgTAG1*	PgTAG1-G1	CAAGAGAGATCTCACCACAA
		PgTAG1-G2	GACAAGTGACTTTCTGCAAG
Phygri07g005850	*PgTAGL1*	PgTAGL1-G1	GGTATATGTGTGCAGTGTTA
		PgTAGL1-G2	TGTTTCTGAAGCCAACACTC
Phygri07g015380	*PgLIN*	PgLIN-G1	TATGGAACAGAATAGCTACC
		PgLIN-G2	AAGTCCTACAAGAGTCTCAA
Phygri12g018340	*PgRIN*	PgRIN-G1	TGAACAACTTGAACGTCAAT
		PgRIN-G2	GAGGCAAATAAGATCAACAA
Phygri03g020760	*PgEJ2*	PgEJ2-G1	CTTATCATCTTCTCTAATCG
		PgEJ2-G2	ACGTTGCAGCTATGCTACTC
Phygri02g012900	*PgTM6*	PgTM6-G1	AATCTGATGATGGTGAAACA
		PgTM6-G2	TGAGAACTCAACAAACAGGC
		PgTM6-G3	TCATTATGCTCTCCAGCACC
		PgTM6-G4	AGATAACATACGTAGTGTTG
Phygri01g009190	*PgGLO1*	PgGLO1-G1	AAAGAGATAAAAAAAAAATG
		PgGLO1-G2	TGGGATCTTGAAAAAAGCAA
		PgGLO1-G3	TAAGACAACATACGGAGTAG
		PgGLO1-G4	ATTGTGGGATGCTAAGCATG
Phygri06g017940	*PgGLO2*	PgGLO2-G1	ACAGTATGCAAGTTAAGCTC
		PgGLO2-G2	TGATGTACAAGTATGTGTGC
		PgGLO2-G3	ACAGTCGGAGATCTTAAGGA
		PgGLO2-G4	AATCCAGGACCAGATTCTGG
Phygri11g018450	*PgDEF*	PgDEF-G1	AAAATAACTTACGAGATAGA
		PgDEF-G2	GATCTGTACCAAAAGACTGT
		PgDEF-G3	AGAGCAGTTGAGGAAGCTAA
		PgDEF-G4	AATCAGGTAGATACTGACCT
Phygri09g010120	*HUSKLESS*	PgHU-G1	AGAGCAAGATATCCCGACGT
		PgHU-G2	GAATAACGAGTTCGTGACCA
		PgHU-G3	CGTCGCCGGAGAAGAAACTG
		PgHU-G4	GATCTATCGGCGAATTACGG

Phygri04g010290	*PgAN1*	PgAN1-G1	GAGAGATGGATATTACAACG
		PgAN1-G2	TAGAAAGACTGTGCAGCCAA
		PgAN1-G3	CGGCTGATTTGAGTCGCCGG
		PgAN1-G4	AGGACCTGACGGAATCCGAG

Supplemental Table S10. Primers used in this study.

**Primers for CRISPR genotyping**				
***P. gri* gene ID**	**gene name**	**primer name**	**primer sequence 5' to 3'**	**Notes**
Phygri12g018350	*PgMPF3*	Pg.MPF3_F1	CTGTCTCTCTACCTTAAAAATTACATC	Related to Figure 3. For genotyping and sequencing CRISPR mutations
		Pg.MPF3_R1	GAGACAATAAACTTGGAAAGTCA	
Phygri11g023460	*PgMPF2*	Pg.MPF2_F1	AGCTCTCCAATATAATATCATCC	Related to Figure 3. For genotyping and sequencing CRISPR mutations
		Pg.MPF2_R1	TCACCAAAGTTCATACTATCTG	
		Pg.MPF2_F2	CACATCCATGTGGATGATAGATC	
		Pg.MPF2_R2	TGGTAGTCAGGTTGTATTCGCC	
		Pg.MPF2_F3	CAATATCTACGTTTACTCAAGCG	
		Pg.MPF2_R3	GCGCGAGTTAATGCAGACAC	
Phygri02g019350	*PgTAG1*	Pg.TAG1_F1	GGATTCTAATCCAAGTTCTTTAGC	Related to Figure 4. For genotyping and sequencing CRISPR mutations
		Pg.TAG1_R1	AGATTAGAACTCTCCGTAACCA	
Phygri07g005850	*PgTAGL1*	Pg.TAGL1_F1	GAGCTATCCTTCTGTAAGCA	Related to Figure 4. For genotyping and sequencing CRISPR mutations
		Pg.TAGL1_R1	CCAAGTATCATAGAGTCATCGAA	
Phygri07g015380	*PgLIN*	Pg.LIN_F1	TCTGTACACTGAGAGCATAAATT	Related to Figure 4. For genotyping and sequencing CRISPR mutations
		Pg.LIN_R1	ACATGTTTACCTGTTCAAAATTAG	
Phygri12g018340	*PgRIN*	Pg.RIN_F1	ATATAGGACCAAAAATAACTATTG	Related to Figure 4. For genotyping and sequencing CRISPR mutations
		Pg.RIN_R1	GGCGGATACATTTGTTTGCAGCA	
Phygri03g020760	*PgEJ2*	Pg.EF2_F1	ACCCACCTTGCACTGGAAAA	Related to Figure 4. For genotyping and sequencing CRISPR mutations
		Pg.EJ2_R1	TCAAAATGTCCTTTAATCTTGTAGC	
Phygri02g012900	*PgTM6*	Pg.TM6_F1	AACTTCATCACTAGAGACAGTGC	Related to Figure 4. For genotyping and sequencing CRISPR mutations
		Pg.TM6_R1	GTACTCTGATATTGATCTATCATCTT	
Phygri01g009190	*PgGLO1*	Pg.GLO1_F1	ATATACTTACCACAATTTTCATCTCA	Related to Figure 4. For genotyping and sequencing CRISPR mutations
		Pg.GLO1_R1	AGAATAAGGTAAAAGAAAAGATGGAAT T	
Phygri06g017940	*PgGLO2*	Pg.GLO2_F1	ATGAGATTAATACAGAACTTGAGCA	Related to Figure 4. For genotyping and sequencing CRISPR mutations
		Pg.GLO2_R1	CTTGGCACTGATGCTAGAAAG	
Phygri11g018450	*PgDEF*	Pg.DEF_F1	CCAAACAAACAGGCAAGTGA	

		Pg.DEF_R1	CCAAGTTTGAACATGAAGTGTGTA	Related to Figure 4. For genotyping and sequencing CRISPR mutations
		Pg.DEF_F2	GTTGAACTGTATGGACTTCCTAT	
		Pg.DEF_R2	ATAAGCTTCAGAGAATCGTCCA	
Phygri09g010120	*HUSKLESS*	Pg.HU_F1	TTGAGTTGATTTTTGTAGCTGA	Related to Figure 5. For genotyping and sequencing CRISPR mutations
		Pg.HU_R1	TTCACTACTGCTCTCTGCTG	
Phygri04g010290	*PgAN1*	Pg.AN1_F1	TGGTACCGTATGTCAATATCA	Related to Figure 2. For genotyping and sequencing CRISPR mutations
		Pg.AN1_R1	GATGCCCGGAGGAAAAGAAA	
	Cas9	35S-seq-F	CTGACGTAAGGGATGACGCAC	For genotyping Cas9 transgenes
		Cas9-R	CATCTCATTACTAAAGATCTCC	
**primers for RT-PCR and qPCR**				
***P. gri* gene ID**	**gene name**	**primer name**	**primer sequence 5' to 3'**	
Phygri04g010290	*PgAN1*	Pg.AN1_RT_F2	GTGGAGAGATGGATATTACAACG	Related to Figure 2E, for RT-PCR and sequencing of *P. gri* and *P. pru* AN1 transcripts
		Pg.AN1_RT_R2	CTGTATTTCCAGCTTGGCTAACA	
Phygri09g010120	*HUSKLESS*	HU_RT_F1	AGAAGCAACAGCAACAGCAG	Related to Figure 5E, for RT-PCR and sequencing of *hu*
		HU_RT_R2	AACAATCAACTTCCATACCCTTCCC	
***S. lyc* gene ID**	**gene name**	**primer name**	**primer sequence 5' to 3'**	
Solyc07g055920	*TAGL1*	qTAGL1_F	AAAAGAGGGAGATTGAGCTGC	Related to Figure 4A
	TAGL1	qTAGL1_R	CTCTACCTCTGCTATCTTTGCG	Related to Figure 4A
Solyc01g056940	*UBI*	qUBI_F	CGTGGTGGTGCTAAGAAGAG	Related to Figure 4A
	UBI	qUBI_R	ACGAAGCCTCTGAACCTTTC	Related to Figure 4A
