# Supplementary material for: Establishing Physalis as a Solanaceae model system enables genetic reevaluation of the inflated calyx syndrome
Source: Plant Cell. 2022 Oct 21;35(1):351–68. doi: 10.1093/plcell/koac305 (PMC9806562; doi:10.1093/plcell/koac305)
Supplement: koac305_Supplementary_Data [file tpc22.00728.PeerReviewReport.pdf › SupplementaryFile/tpc22.00728.PeerReviewReport.pdf]

Establishing *Physalis* as a *Solanaceae* model system enables genetic reevaluation of the inflated calyx syndrome

Jia He, Michael Alonge, Srividya Ramakrishnan, Matthias Benoit, Sebastian Soyk, Nathan T. Reem, Anat Hendelman, Joyce Van Eck, Michael C. Schatz, Zachary B. Lippman

Corresponding author: Zachary B. Lippman, lippman@cshl.edu

Review timeline:

**TPC2022-RA-00728**	Submission received:	July 28, 2022
	1st Decision:	Sept. 3, 2022 *minor revision requested*
**TPC2022-RA-00728R1**	1st Revision received:	Sept. 18, 2022
	2nd Decision:	Sept. 22, 2022 *acceptance pending, sent to science editor*
	Final acceptance:	Oct. 11, 2022

REPORT: (The report shows the major requests for revision and author responses. Minor comments for revision and miscellaneous correspondence are not included. The original format may not be reflected in this compilation, but the reviewer comments and author responses are not edited, except to correct minor typographical or spelling errors that could be a source of ambiguity.)

TPC2022-RA-00728 1st Editorial decision – *minor revision requested* **Sept. 3. 2022**

We have received reviews of your manuscript entitled "Establishing Physalis as a new Solanaceae model system enables genetic reevaluation of the inflated calyx syndrome." On the basis of the advice received, the board of reviewing editors would like to accept your manuscript for publication in The Plant Cell. This acceptance is contingent on revision based on the reviewer comments. Each of the three reviewers requests minor clarifications. In addition, reviewer #1 suggests a cross of P. pruinosa and the P. grisea AN1 CRISPR knock-out line i(PgAN1) to verify that the anthocyanin phenotype won't be rescued and PprAN1 loss of function is responsible for the loss of anthocyanin in P. pruinosa. I agree with the reviewer that the suggested cross would be a nice addition, if it can be done without too much fuss. Otherwise, it would be acceptable to simply acknowledge that it is possible that another anthocyanin pathway gene or a regulatory linked to AN1 may be responsible for loss of flower color in P. pruinosa. All other reviewer comments/suggestions can be addressed with minor revisions to the text.

-- Reviewer comments:

[Reviewer comments shown below along with author responses]

TPC2022-RA-00728R1 1st Revision received **Sept. 18, 2022**

Reviewer comments and **author responses**:

Reviewer #1:

In this manuscript, He et al. presented chromosome-level genome assemblies of two groundcherry species, identified the causal gene responsible for the most obvious phenotypic difference between the two (presence vs. absence of anthocyanin pigments in the petal nectar guide and stem), generated CRISPR knockout mutants for 11 MADS-box genes that are potential candidates for controlling the Inflated Calyx Syndrome (ICS), and performed a proof-of-principle EMS mutagenesis to demonstrate the power of "traditional" forward genetics in unbiased dissection of the real genetic network regulating ICS.

Both the quantity and the quality of data presented here are very impressive. By the end of the manuscript, I was completely convinced by the tremendous potential of this system in studying the genetic origin of a novel trait (ICS) in Solanaceae and in comparative genomic and functional analysis across this super important family.

It was a pity that none of the 11 MADS-box genes, including the ones claimed to be essential regulators of ICS in previous studies, turned out to affect ICS. However, I think it's actually quite important to publish these "negative" results, as that will help clear the mess and re-orient the search for real answers.

I enjoyed almost all aspects of this paper and have only one slightly major comment on the AN1 gene and a few minor comments on the writing.

On the conclusion that AN1 is the causal gene underlying the anthocyanin variation between P. grisea and P. pruinosa: Although the co-segregation between the phenotype and the intron-retention pattern of AN1, and the CRISPR phenotypes are appealing evidence, stronger evidence could come from a complementation cross between the PgAN1 CRISPR line (after crossing out the CAS9 transgene) and P. pruinosa. Loss of anthocyanin pigmentation by knocking out PgAN1 per se does not prove AN1 is the causal gene between the two natural species. For example, knocking out any of the anthocyanin biosynthesis genes on the pathway shown in Figure 2C (e.g., CHS, CHI, FSH, DFR, ANS) in P. grisea will probably also lead to loss of anthocyanin pigmentation, but that doesn't mean they are all causal genes for the natural variation. Also, a sentence or two describing the phylogenetic context here would be helpful. It might be obvious to the authors that the difference between P. grisea and P. pruinosa is due to LOSS of anthocyanins in P. pruinosa, but for readers who are not familiar with the Physalis phylogeny, there is no way for us to tell immediately whether it's a LOSS in P. pruinosa or GAIN in P. pruinosa. If the complementation cross can be done within 3 months, I would suggest the authors to incorporate the complementation results. However, if this experiment will take significantly longer, I don't think it's necessary to wait for this; perhaps just acknowledge in the text that further complementation crosses or rescue experiments can enhance the conclusion.

Response: We appreciate the reviewer's positive comments on our manuscript, especially recognizing the importance of our data showing that the 11 MADS-box genes we tested do not impact ICS, despite what has been published over the last two decades. In fact, as described below in response to a shared reviewer comment on possible functional redundancy, we are pleased to present CRISPR double mutants of the proposed key regulators *MPF2* and *MPF3*. Notably, these new *mpf2 mpf3* mutant plant also maintain ICS (shown in expanded Fig. 2), thereby enhancing and reinforcing our conclusions.

Thanks for pointing out that a complementation test would definitively conclude the causal relationship between the *AN1* SV and the pigmentation differences. While we are making efforts to perform the complementation test, it would significantly delay publishing the manuscript. We appreciate both reviewer 1 and the editor recognizing this is not an essential experiment for publication. Thus, we have acknowledged in the manuscript that it is possible, though highly unlikely, that another variant linked to *AN1* could be responsible for the color variation. As for the phylogenetic context of anthocyanin production, we are not aware of any literature specifically studying this in *Physalis*. From our growing of different species of *Physalis,* it appears purple guides is the ancestral state. While a much larger sampling of *Physalis* species could elucidate evolutionary context of pigmentation/anthocyanin production, we prefer not to deviate from the main points of the manuscript. Indeed, in the manuscript, we used this trait variation as a first demonstration of the utility of these systems in dissecting natural variation through forward genetics. To simplify this message and take it out of the context of phylogeny, we changed the text from: "underlies the <u>loss</u> of pigmentation in *P. pruinosa*" to "underlies the <u>absence</u> of purple pigmentation in *P. pruinosa*".

Minor linguistic suggestions:

Page 3. First paragraph of INTRODUCTION: perhaps change "species-specific limitations" to "species-specific limitations of the other taxa"; otherwise it reads like the limitations of tomato have made tomato the preferred model.

Response: Thank you for the suggestion. We have changed the language to make it clear.

Page 3. The end of the second paragraph: "also found also".

Response: We have corrected the typo.

Page 7. Third paragraph: "four orthologs of anthocyanin pathway genes on chromosome 4". Do you mean ""four orthologs of anthocyanin pathway genes and their regulators on chromosome 4"? "Anthocyanin pathway genes" usually mean the structural genes that encode enzymes.

Response: Thank you for the suggestion. We changed the text to "four orthologs of anthocyanin pathway genes

and their regulators on chromosome 4".

Reviewer #2:

The manuscript, Establishing Physalis as a new Solanaceae model system enables genetic reevaluation of the inflated calyx syndrome" by He et al. reports the genome of two Physalis species and investigates the genetic basis of petal color variation and the inflated calyx syndrome (ICS). The data presented in the manuscript is of interest to a broad audience interested in plant evolution and development and the analyses are sound and comprehensive.

Major points:

1. There is just one major point that I would like the authors to address: If I am not wrong, all MADS-box-gene mutants studied were single mutants. In my opinion, it is not unlikely that double mutants may perturb ICS. This would also fit to the observed phenotypes of RNAi mutants as two closely related genes may be knocked-down in such a case. Concordantly, the authors state in the discussion "This might suggest double and higher order mutants ... would ultimately perturb ICS, possibly reflecting a collective role of multiple family members acting redundantly or in a network. However, such a result would not necessarily indicate direct roles for these genes in the evolutionary steps leading to ICS." However, I do not fully get this argument. Could the authors be more precise on the possible role of redundancy between MADS-box genes for the ICS?

Response: Thank you for these comments. As MADS-box proteins are involved in myriad aspects of reproductive development, mediated by entangling physical interactions and complex feed-back regulation, it might be expected that mutations in individual genes (especially given published data on *MPF2* and *MPF3*) would disrupt ICS. Lending more support to the roles of single genes is the many examples of MADS-box gene overexpression causing a mimic of inflation Thus, our initial hypothesis was that mutations in at least one of our chosen 11 MADS-box genes, especially *MPF2* and *MPF3,* would have had at least had some effect on ICS. As the reviewer notes, we agree with the possibility that combinations of MADS-box-gene mutations (not limited to genes and mutants in our study) may ultimately perturb ICS. However, our main point, which we feel is clear, is that even if MADS-box redundancy is found in future work to control ICS, this would not necessarily indicate a primary involvement in the evolutionary trajectory leading to the emergence of ICS, as has been suggested multiple times in the literature, particularly for *MPF2* and *MPF3.*

The idea of creating many double and higher-order mutations of the MADS-box genes we targeted is attractive, and could be a fast-track to achieving perturbation of ICS. However we feel this approach is prone to many genetic and phenotypic challenges, especially regarding the highly pleiotropic roles of these genes in meristem development/maturation, flowering transition, inflorescence architecture and floral identity. We recognize, however, that our initial submission lacked the obvious experiment of generating *Pgmpf2 Pgmpf3* doubles mutants. We are pleased to include in our revision this key result in a revised Figure 3.

Notably, double mutants of *mpf2 mpf3* showed normal ICS. Thus, at least for these MADS-box genes, long suggested as *causative* in the evolution of ICS, redundancy is not the explanation. This result not only reinforces the main findings of our study, but also further supports our proposal that the search of factors underlying ICS requires a fine dissection of sepal transcriptomes throughout inflation to elucidate the molecular events of ICS in an unbiased way before unraveling the evolutionary steps.

Minor points:

1. It is mentioned several times in the manuscript that Physalis occupies a key phylogenetic position. Please explain what is so special about the phylogenetic position.

Response: *Physalis* is a representative genus of Physaleae, an important tribe of Solanaceae capturing large generic diversity, which is under-studied and with limited genomic resources. Moreover, recent discoveries of Physaloid fruiting fossils *(Physalis infinemundi* and *Physalis hunickenii*) dated to ~52 Ma, in contrast to previous phylogenetic framework of Solanaceae estimated the origin of Physalideae 16.7–24 Ma. In comparative studies within Solanaceae, *Physalis* could potentially capture diversification over long evolutionary distance. We appreciate the comment to make this more clear, and we have made changes to the text to capture this point.

2. In the results, the authors state "These results indicate that the SV in P. pruinosa AN1 (PprAN1) underlies the loss of pigmentation in P. pruinosa". However, the causative difference could still be in a regulator of AN1 on chromosome 4, couldn't it?

Response: This is a fair point. Our mapping interval was indeed large, but still allowed us to focus our search for candidate genes and variants to a much smaller region of the genome. Even more, we note that the few other candidate genes in the anthocyanin pathway on chromosome 4 have no obvious mutations to explain such a strong phenotype. All of this in comibation with our CRISPR results, strongly suggests *AN1* is the responsible gene. However, both reviewer 1 and this reviewer are fair to ask for a complementation test between our CRISPR mutants of *AN1* in *P. grisea* and the natural *AN1 SV* mutation in *P. pruinosa* to unequivocally demonstrate this gene is causative. Though attempts at making this cross are ongoing, this experiment would take more than three months to demonstrate what we feel will be a minor addition to what is already a highly likely conclusion – that *AN1* and this specific SV affecting splicing and causing premature stop codon – is responsible. As noted in the response to reviewer 1's comments, we have noted in the revision that it is possible, though highly unlikely, that another variant linked to *AN1* could be responsible for the color variation.

3. Are there any phenotypic effects of the Pgmpf2CR mutation?

Response: All alleles of the *Pgmpf2CR* mutants appear wild type in terms of calyx and general morphology in our normal greenhouse and field conditions. We cannot rule out the possibility of phenotypic effects under other conditions, but exploring such effects, though potentially interesting from a broader developmental and comparative evolution perspective, goes beyond the scope of making this specific mutant, which was to test a role in ICS.

4. When it is mentioned in the manuscript that "Physalideae ... ICS was gained multiple times", I think it would be important to note that it was only gained once in the Physalis genus (if I am not mistaken).

Response: Very good point. We have made changes to the text to make this distinction clear.

5. To which MADS-box-gene family does MBP21/JOINTLESS-2 (J2) belong to?

Response: *J2* belongs to the type II (MIKC) MADS-box-gene family, and is in the *SEPALLATA4* (*SEP4*) clade, together with *EJ2*, *LIN* and *RIN*. This was stated in the main text, but we have now brought this context earlier when *J2* is first mentioned.

6. The third sentence of the abstract is very long. Please split the sentence into two for better readability.

Response: Thanks for the suggestion. We also recognized the length of this sentence, but ultimately decided the flow worked better to capture these points in one sentence. We would be happy for editorial suggestions on how to split this sentence, but we prefer to keep it as is.

Reviewer #3:

Jia He and co-authors assembled two high-quality Physalis genomes, and constructed the genetic editing system in Physalis, which lay the foundation for it to be another potential model system, in addition to tomato and petunia. They also used genetic editing of candidate genes coupled with genetic mapping to rule out and narrow down the causal gene for the inflated calyx syndrome in Physalis. The resources and findings of this study are of values and interests to close related research fields and those who are interested in fruit development. Here I have several concerns for the authors to improve the ms.

1. Please introduce a bit about the previously sequenced genome of Physalis in the Introduction section, and its relationship to the two currently assembled genomes. In the Results section, why not show the syntenic gene of Ben in this reported physalis genome in the synteny genomic analysis of this ms.?

Response: We thank the reviewer for the suggestions. In the original manuscript (and in Supplementary tables), we included quality comparisons of the previous genome and our genomes. A more detailed comparative analysis of those genomes would not provide much useful information regarding the main points of this study. Even more, we respectfully disagree with some of their major conclusions, which could have resulted from a lesser quality genome

compared to ours. Thus, we decided not to discuss more of the other genome, in order to prevent distraction from our main message.

For the Ben and Bob synteny, we checked the other genome, and similarly, the Bob ortholog is absent. We respectfully feel it is unnecessarily to include this information in the revised manuscript, as adding a third syntenic region does not change our conclusion based on our own two high quality genomes, and would simply point out that, at least for this region, the previously published genome produced a syntenic region that matches our own. Beyond this, we have already mentioned (lightly) that the previous genome is of much lower quality compared to our two genomes. Adding this syntenic region to our figure would potentially imply that the previous genome is of sufficient quality to match ours as a reference, which it does not.

2. The first two paragraph, especially the first paragraph of the Results "Chromosome-scale reference genomes of P. grisea and P. pruinosa" are not contents of results, thus should be largely moved to the Introduction section.

Response: All of the authors on the manuscript had discussions and contributed to revisions regarding this point before submitting the original manuscript. All of us felt that due to the content and structure of this study, moving these paragraphs to the introduction would affect the flow and readability of the manuscript significantly. We respectfully decline making this change.

3. The first sentence of the third paragraph, I see no information that support "a key phylogenetic position among Solanaceae model systems". You need some comparisons with other solanaceae species to get this sentence, other than just describing the features of Physalis itself.

Response: Please see response to reviewer 2, Minor points 1.

4. In the part "A structural variant in the bHLH transcription...", the second and third sentences in the paragraph two, as well as the first sentence in the paragraph three should be deleted or largely shorten, related figures can also be deleted, as this information is well known, thus can be simply referred by citing related papers.

Response: We thank the reviewer for the suggestion. In the original manuscript, we aimed to achieve the right balance of being succinct and informative about the background of anthocyanin and pigmentation to general audience. We respectfully have decided to keep this text as it is currently written, but we appreciate the suggestions for alternative writing styles.

5. I suggest to merge the two parts of "The MADS-box genes MPF2..." and "Targeted mutagenesis of additional..." in the Results section.

Response: These two parts are intended to be separate as our logic flow transitions from "re-evaluating the previously proposed key regulators" to "using reverse genetics to interrogate new candidates". As above, we respectfully have decided to keep the current structure.

6. The first two paragraph of the Results "The MADS-box genes MPF2..." should be largely shorten and merged with the third paragraph, as they are not results. Contents on discussion should be referred as concise as possible in the Results section.

Response: We appreciate the reviewer's perspective on writing structure and style, but after many revisions involving many suggestions from authors and others, all agreed that the current text structure is preferred, as we felt this small amount of background and redundancy in this particular section would help with the logic, flow, and readability of the text.

7. As the authors discussed, multiple MADS-box genes might function collectively as a complex network or function redundantly in the regulation of ICS. I wonder whether it is possible to double/triple-mutate those genes sharing high sequence/function similarities based on previous reports and also the phylogenetic relationships. I know that many MADS-box genes are derived from the same ancestral gene, and were duplicated and diversified through WGD/tandem duplication. Still, taking the phylogenetics into consideration might provide new perspective to this issue.

Response: We appreciate the suggestion of the reviewer. Please see response to reviewer 2, Major point 1.

8. Do Ben and Bob belong to a tandem gene array? Please specify it clearly.

Response: According to the original study, the petunia *BEN* and *BOB* were extracted from fragmented genome assemblies; however, double mutants were easily obtained from the cross of the two single mutants, so they are clearly not tightly linked. Moreover, the tomato orthologs of *BEN* and *BOB* are located on Chr9 and Chr10 respectively, which was reinforced through our synteny analysis with our *Physalis* genomes. We do not feel any clarification in the text is needed for this, as our results show *BEN* and *BOB* orthologs are on different chromosomes.

9. For the conclusion "...how lineage-specific gene duplications and paralog presence-absence variation...", I can't see direct results or analysis associated with or support this conclusion. Please make related results/evolutionary analysis (more?) clearly and carefully.

Response: We tuned the text to "Our identification of *huskless* through forward genetics exemplifies how paralog presence-absence variation can shape genetic redundancies and genotype-to-phenotype relationships, and further illustrates the value of multiple related model systems."

10. For the 60,087 homozygous SNPs, please provide the information of how many coding and nonsynonymous SNPs out of these 60,087 homozygous SNPs, before introducing the 43 genes with high impact.

Response: We now have updated Supplemental Table 3 to Supplemental Table 3a and 3b to include the information requested by the reviewer.

TPC2022-RA-00728R1 2nd Editorial decision – *acceptance pending* **Sept. 22, 2022**

We are pleased to inform you that your paper entitled "Establishing Physalis as a new Solanaceae model system enables genetic reevaluation of the inflated calyx syndrome" has been accepted for publication in The Plant Cell, pending a final editorial review by a science editor.

Final acceptance from Science Editor **Oct. 11, 2022**
